# Nonreciprocal metasurface with space–time phase modulation

**DOI:** 10.1038/s41377-019-0225-z

**Published:** 2019-12-18

**Authors:** Xuexue Guo, Yimin Ding, Yao Duan, Xingjie Ni

**Affiliations:** Department of Electrical Engineering, The Pennsylvania State University, University Park, PA 16802 USA

**Keywords:** Metamaterials, Sub-wavelength optics

## Abstract

Creating materials with time-variant properties is critical for breaking reciprocity that imposes fundamental limitations on wave propagation. However, it is challenging to realize efficient and ultrafast temporal modulation in a photonic system. Here, leveraging both spatial and temporal phase manipulation offered by an ultrathin nonlinear metasurface, we experimentally demonstrated nonreciprocal light reflection at wavelengths around 860 nm. The metasurface, with travelling-wave modulation upon nonlinear Kerr building blocks, creates spatial phase gradient and multi-terahertz temporal phase wobbling, which leads to unidirectional photonic transitions in both the momentum and energy spaces. We observed completely asymmetric reflections in forward and backward light propagations over a large bandwidth around 5.77 THz within a sub-wavelength interaction length of 150 nm. Our approach highlights a potential means for creating miniaturized and integratable nonreciprocal optical components.

## Ultrathin and dynamic: Controlling the flow of light

Light propagating in one direction only could pave the way towards miniaturized components for faster, more efficient data transfer in optical circuits. Xingjie Ni and colleagues at the Pennsylvania State University demonstrated the unidirectional flow of near-infrared light in free space based on a 150-nanometre-thick nano-engineered surface, i.e., metasurface. Their approach adds to the body of research examining ways to disrupt the common wisdom that light travelling in one direction can also travel in the other. In the new system, the ultrathin metasurface consisting of a silver reflector plate supporting block-shaped, silicon nanoantennaswas dynamically modulated to generate time-varying parameters. The infrared light interacts with the ultrathin metasurface, ultimately resulting in a reflection that does not propagate in the opposite direction.

## Introduction

Reciprocity is a fundamental principle rooted in linear physical systems with time-reversal symmetry, requiring that the received–transmitted field ratios are the same when the source and detector are interchanged^1^. However, it is preferable to break reciprocity in many practical applications, such as lasers and full-duplex communication systems, so that back-scattering from defects or boundaries can be avoided^2^. Traditionally, nonreciprocity has been realized through magneto-optic materials that are too bulky and lossy to be integrated into modern photonic systems^3^. In addition, nonlinear materials^4–6^ are employed to achieve nonreciprocity at the cost of a high intensity requirement, but they suffer from poor isolation and are reciprocal to noises^7^. To circumvent these limitations, an increasing number of studies have focused on developing materials with time-variant properties in which time-reversal symmetry is explicitly broken to achieve nonreciprocity^1,8^. To date, based on the strong electro-optic^9,10^, acousto-optic^11–14^, or optomechanical effects^15,16^ of different materials, proof-of-concept temporal modulation has been demonstrated at frequencies ranging from kilohertz to gigahertz, which are much lower than the optical frequency as a result of the slow carrier injection of electro-optic modulation and low-frequency acoustic or mechanical modes in acousto-optic or optomechanical modulation. In addition, these dynamic systems suffer from limited bandwidth either due to the group velocity mismatch among photonic modes or the intrinsic narrow linewidths of acoustic and mechanical modes. Moreover, they require long interaction lengths to observe the desired effect. Nonreciprocity with a sub-wavelength interaction length and an ultrafast modulation frequency over the THz bandwidth is technically challenging and has not been realized to date.

**Fig. 1:**
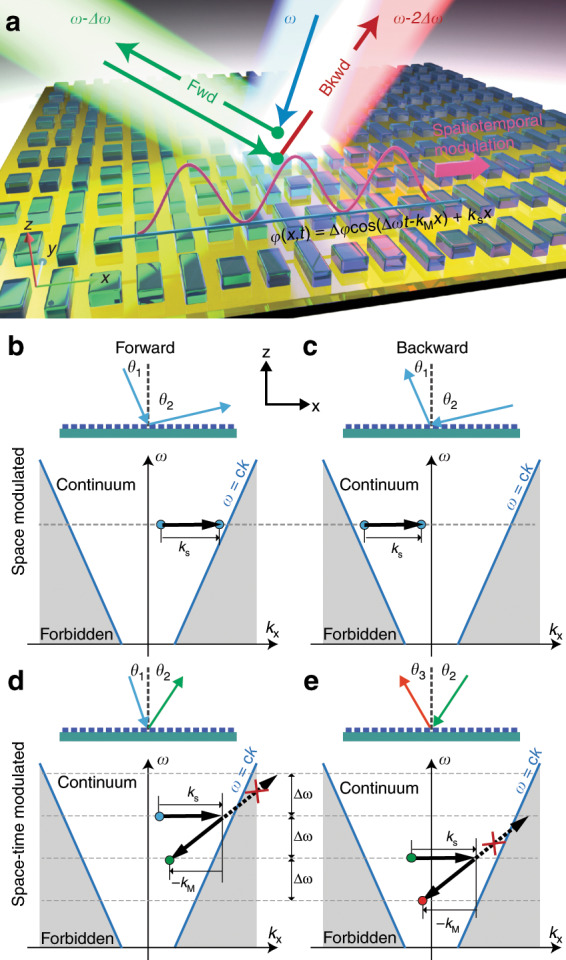
Working principle of a nonreciprocal space–time phase modulated metasurface. **a** An illustration showing the concept of a space–time phase modulated metasurface consisting of resonating dielectric nanoantennas operating in reflection mode. A travelling phase modulation in sinusoidal form is superposed on the designed phase gradient along the x direction. Light impinging on the metasurface with frequency *ω* is converted to a reflecting beam with frequency *ω* – Δ*ω* due to the parametric process arising from dynamic phase modulation, while the back-propagating beam with frequency *ω* – Δ*ω* is converted to *ω* – 2Δ*ω* instead of *ω*, resulting in a nonreciprocal effect. **b**, **c** and **d**, **e** Comparison between a regular space modulated metasurface (**b** and **c**) and a space–time phase modulated metasurface (**d** and **e**). A regular space-modulated metasurface supports only symmetric forward (**b**) and backward (**c**) reflections, as shown in the dispersion diagrams. There is no frequency conversion (i.e., no transition in the energy space). The process is reciprocal, and the forward and backward beams share the same trajectory. In contrast, a space–time phase modulated metasurface supports asymmetric forward (**b**) and backward (**c**) reflections. It not only offers additional momentum along the x direction to the reflected light but also converts its frequency. In either the forward or backward case, the upward transition is forbidden because the resulting wavevector will be too large to be supported in free space, resulting in unidirectional photonic transitions in both the energy and momentum spaces. Therefore, the trajectories of beams differ and reveal nonreciprocity effects. *k*_s_ is the linear momentum introduced by the spatial phase modulation, and *k*_M_ is the additional linear momentum introduced by the temporal phase modulation.

Here, we experimentally demonstrate a new approach that achieves nonreciprocity on an ultrathin metasurface with space–time phase modulation. A metasurface^17^ is an optically thin nanostructured two-dimensional material that can manipulate light through its subwavelength-sized building blocks. Designed specifically to achieve a controlled optical response, metasurfaces have been used to create novel optical devices, including invisibility cloaks^18^, flat lenses^19–21^, and ultrathin holograms^22,23^, to enhance nonlinear generation^24,25^, and to explore interesting physical effects^26,27^. While these concepts have opened up a new paradigm for manipulating light with an ultrathin layer, there are fundamental limitations that a spatially modulated metasurface alone cannot overcome. In particular, a time-varying response is required to violate reciprocity in a non-power and non-magnetic-field-dependent fashion. Metasurfaces with spatiotemporal modulation^28^ in the index^29,30^ or directly in their phase profile^31^ have been theoretically proposed recently and experimentally demonstrated at radio frequencies^32–36^, offering an opportunity to overcome this limitation. We experimentally demonstrate this new dynamic metasurface with an additional fast temporal phase modulation. Our space–time modulated metasurface (Fig. 1a) consists of a set of specifically designed nonlinear nanoantennas that not only provide abrupt static phase shifts for the incident light but also are capable of changing the phase shifts dynamically in the presence of an external travelling-wave modulation. To introduce the dynamic phase change to the metasurface, we incorporate a heterodyne interference of two coherent waves to generate multi-terahertz (~2.8 THz) time-varying modulation^37^. This breaks the time-reversal symmetry of the metasurface. Our experiments demonstrate nonreciprocal light propagation in free space at around *λ* = 860 nm across an ultrathin (150 nm) layer. Furthermore, we achieve completely asymmetric photonic transitions over a wavelength range of ~15 nm (corresponding to a bandwidth of 5.77 THz). We believe that the space–time modulated metasurface demonstrates a viable way to obtain ultrafast time-variant material responses and potentially motivates a new direction in constructing miniaturized and integratable magnetic-free nonreciprocal optical components.

## Results

A conventional spatially modulated metasurface with a phase gradient (e.g., *φ* = *k*_s_*x*) on the surface is capable of imparting additional linear or orbital angular momentum to incident light, which breaks the inversion symmetry and enables full control over the photonic transitions in the momentum space. However, this process is linear and time-reversal symmetric and is inherently reciprocal (Fig. 1b, c). In contrast, our space–time modulated metasurface has a phase modulation of the form

1 $$\varphi (x,t) = \Delta \varphi \cos (\Delta \omega t - k_{\mathrm{M}}x) + k_{\mathrm{s}}x$$

where Δ*φ* is the temporal modulation depth, Δ*ω* the modulation frequency, *k*_M_ is the modulation spatial frequency, and *k*_s_ is the static phase gradient introduced by the spatial distribution of the nanoantennas. This modulation acting upon the incident light field gives an additional space–time varying phase factor, expressed as exp[*iφ*(*x*,*t*)]. Applying the Jacobi–Anger expansion, this phase term can be rewritten as a series of Bessel functions of the first kind, which enables the reflected field to be expressed as

2 $${\vec E_{\mathrm{r}}}\left( {\vec r,t} \right) = \zeta J_0\left( {\Delta \varphi } \right)\vec E_{\mathrm{i}}e^{i\left( {\vec k_{\mathrm{i}}\vec r + k_{\mathrm{s}}x - \omega _{\mathrm{i}}t} \right)} + i\zeta J_1\left( {\Delta \varphi } \right)\vec E_{\mathrm{i}}\left\{ {e^{i\left[ {\left( {\vec k_{\mathrm{i}}\vec r + k_{\mathrm{M}}x + k_{\mathrm{s}}x} \right) - \left( {\omega _{\mathrm{i}} + \Delta \omega } \right)t} \right]} + e^{i\left[ {\left( {\vec k_{\mathrm{i}}\vec r - k_{\mathrm{M}}x + k_{\mathrm{s}}x} \right) - \left( {\omega _{\mathrm{i}} - \Delta \omega } \right)t} \right]}} \right\}$$

where *ω*_i_ and $\vec k_{\mathrm {i}}$ are the frequency and free-space wavevector of the incident wave, $\zeta = \sqrt \eta$, and *η* is the static diffraction efficiency of the metasurface. Note that only the zeroth- and first-order Bessel functions are retained since the phase modulation depth Δ*φ* is small, which leads to negligible contributions from higher-order functions. It is evident from the second term on the right-hand side of Eq. (2) that a sinusoidal phase component will be decomposed into two photonic transitions (i.e., sidebands) in the energy space with resulting frequencies *ω*_r_ = *ω*_i_ ± Δ*ω*, where ‘ + ’ and ‘–’ denote an upward and downward transition, respectively. The dynamic phase modulation breaks reciprocity and leads to time-reversal-asymmetric photonic transitions^38^. Different from the recently reported phonon-assisted nonreciprocal waveguides^13,14^ based on indirect interband photonic transitions^38^, our system is naturally phase matched for all free space modes and does not require complex design of the acoustic and photonic modes to fulfil stringent momentum/energy matching conditions. Furthermore, our space–time metasurfaces exhibit agile control over both momentum and energy conversions. To achieve unidirectional frequency conversion, the metasurface can be designed to either fulfil *k*_ix_ + *k*_s_ + *k*_M_ > *k*_i_ (*k*_ix_ is the projection of incident wavevector *k*_i_ along the x direction), 2*k*_M_ > *k*_i_, and –*k*_i_ < *k*_ix_ + *k*_s_ – *k*_M_ < *k*_i_ to ensure unidirectional down-conversions (Fig. 1d, e) or fulfil *k*_ix_ – *k*_s_ – *k*_M_ < –*k*_i_, 2*k*_M_ > *k*_i_ and –*k*_i_ < *k*_ix_ – *k*_s_ + *k*_M_ < *k*_i_ to ensure unidirectional up-conversions. As an example, we depict the case of unidirectional down-conversions in Fig. 1d, e. The optical paths of forward and backward propagation are shown schematically in the top panel, and the photonic states represented by different colour dots are shown in the bottom dispersion diagrams. The photonic transition from the blue dot state to green occurs in the forward propagation, whereas green to red occurs in the backward propagation. With a space–time modulated metasurface, the frequency transitions arise from the parametric processes caused by the temporal modulation, while the momentum transitions arise from both temporal and spatial modulation. As a result, the allowed transitions (i.e., downward transitions) can be selected by pushing a given output state (i.e., upward transitions) to the forbidden (i.e., non-propagative) region with a unidirectional momentum transfer, *k*_s_, provided by the spatial phase modulation of the metasurface. Note that the reflection angle of the backward propagating light is not necessarily the same as that of the forward propagating light even though they have the same *x* component of the wavevector (*k*_x_), because the frequency shift also changes the length of the wavevector. The paths of the incident and returning beam overlap only in the special case of normal incidence (i.e., *k*_ix_ = 0), which is particularly useful for free-space optical isolators. For completeness, we also presented bi-directional photonic transitions on the space–time metasurfaces, which also exhibit nonreciprocity (Fig. S2). In all cases, the spatiotemporal phase modulation leads to completely asymmetric reflections in the forward and backward directions. The back reflected wave does not return to the same state as the forward incident wave, and therefore, the process is nonreciprocal.

**Fig. 2:**
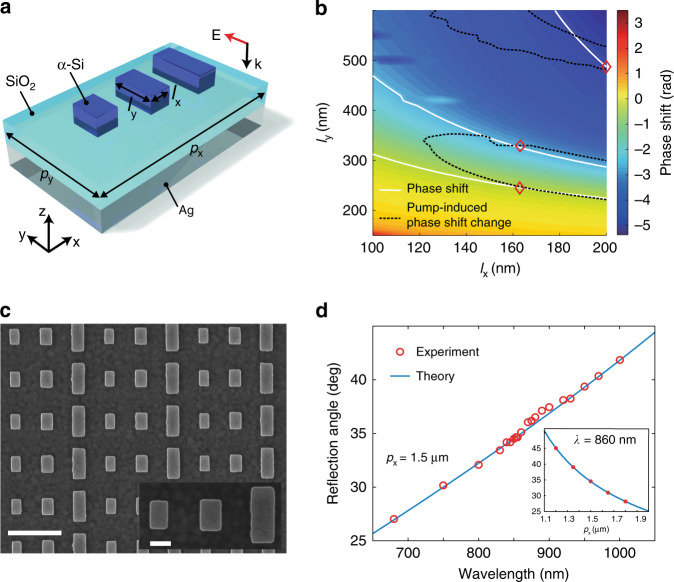
Design and characterization of the metasurface. **a** A 3D illustration of a unit cell of the metasurface, which consists of three α-Si nanobar antennas. The thickness of the silver ground plate, the SiO_2_ spacer layer, and the α-Si nanoantennas is 200, 50, and 150 nm, respectively. **b** Calculated phase shifts (surface plot) of reflected light in a 2D parameter space spanned by *l*_x_ and *l*_y_. It is overlaid by contour lines showing the pump-induced phase shift change of 0.32 radians (black dashed line) when illuminated with pump light at an intensity of 15 GW/cm^2^. The white lines are contours indicating the evenly spaced phase shifts in the static condition. Three different nanoantennas that cover 2*π* static phase shifts with an interval of 2*π*/3 are chosen as the building blocks to construct the metasurface, as marked by the red diamonds intersecting the two types of contour lines. **c** Field emission scanning electron microscopy (FESEM) image of a fabricated α-Si metasurface. Scale bars in the main figure and the inset are 1 µm and 200 nm, respectively. **d** Measured (red circles) and calculated (blue line) anomalous reflection angles of the metasurface at wavelengths ranging from *λ* = 680 nm to 1000 nm at normal incidence, with *p*_x_ = 1.5 µm. The inset shows the anomalous reflection angles for samples with *p*_x_ varying from 1.2 µm to 1.8 µm at *λ* = 860 nm. The experimental results agree well with the theory.

We used a set of nanobar antennas (Fig. 2a) made of amorphous silicon (α-Si), which has a large Kerr index and low optical loss, as the building blocks of the metasurface. With the adoption of a 50-nm-thick SiO_2_ spacer layer and a silver back-reflector plate to create a gap resonance, the nanoantenna can induce a large phase shift (over 2*π*) upon arrival of the incident light. The permittivity of the α-Si nanoantennas can be modulated by an intense optical field due to the nonlinear Kerr effect. This is an ultrafast process (Fig. S6) and is the key to obtaining the THz temporal phase modulation. Subsequently, the light-induced permittivity change of the nanoantennas will detune their resonances and therefore lead to a change in the phase shift upon arrival of the incident light at the operational wavelength. The static phase shifts (*φ*) and the changes in the phase shifts (Δ*φ*) induced by the pump light (*λ*_pump_ = 800 nm, *I*_pump_ = 15 GW/cm^2^) are simulated separately and mapped out for the selection of designs in a two-dimensional parameter space spanned by nanoantenna dimensions *l*_x_ and *l*_y_. Three different nanoantennas with static phase shifts covering a 2*π* range while preserving a uniform phase shift change under pump light illumination were chosen to build a supercell of the metasurface. The parameters for the nanoantenna designs used in our experiment are indicated as red diamonds in Fig. 2b. The spatial distribution of these nanoantennas creates a static phase gradient *k*_s_ = 2*π*/*p*_x_, where *p*_x_ is the supercell period in the x direction. Note that the nanoantennas transform the small permittivity modulation from the Kerr effect into relatively large dynamic phase modulation, leading to an efficient nonreciprocal photonic transition at moderate pump power. Moreover, the local field is enhanced by those resonant elements. As a result, the designed nanoantennas significantly boost the temporal phase modulation depth.

We fabricated the metasurface as shown in Fig. 2c and characterized its linear performance with a *k*-space imaging microscope (Fig. S5a). Our metasurface attains an overall diffraction efficiency of 84% *experimentally* around the designed operational wavelength *λ* = 860 nm (Fig. S5c). The measured reflection angles agree with the theory at different wavelengths and different supercell periods (Fig. 2d).

**Fig. 3:**
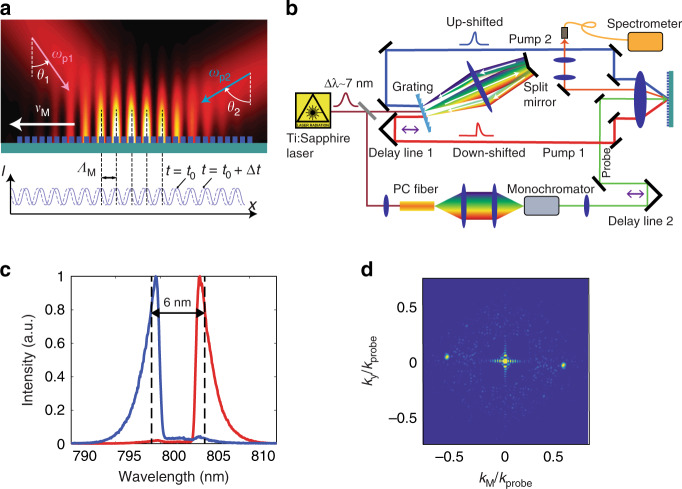
Experimental demonstration of controllable space–time phase modulation. **a** A schematic of two pump beams with closely spaced frequencies *ω*_p1_ and *ω*_p2_ impinging on the metasurface with incident angles *θ*_1_ and −*θ*_2_, respectively. They produce travelling interference fringes with a period of *Λ*_M_ and a speed of *ν*_M_ = Δ*ω*/*k*_M_ on the metasurface. The nanoantennas on the metasurface exhibit a time-variant change in the phase shifts induced by the travelling interference fringes. **b** A schematic of the experimental setup. The output of a Ti:Sapphire femtosecond pulsed laser at 800 nm is split into two beams: one is directed through a transmission grating to generate frequency-shifted pump beams; the other is sent to a photonic crystal fibre (PCF) to create a wavelength-tunable probe beam. Two delay lines are employed to achieve temporal synchronization among the three beams. An aspheric lens focuses pump and probe beams onto the metasurface. The reflected signal is picked up by a D-shaped mirror and detected by a spectrometer. We map the frequency and momentum of the reflected signal by monitoring the collected spectra across the Fourier plane of the aspheric lens. **c** Spectra of the two pump beams, showing a wavelength difference of 6 nm (corresponding to ∆*f* of 2.8 THz). **d** The 2D Fourier transform of the interference pattern of the two pump beams, revealing a *k*_M_ equal to 0.54*k*_probe_, where *k*_probe_ is the free space wavevector of the probe light at *λ* = 860 nm.

Next, we developed a fast temporal modulation technique that uses a heterodyne interference between two laser lines that are closely spaced in frequency (Fig. 3)^39^. In contrast to a homodyne interference setup, the heterodyne interference pattern results in a travelling-wave intensity distribution given by *I*(*x*,*t*) = *I*_0_[1 + cos(Δ*ωt−k*_M_*x*)], where *k*_M_ = 2*π*/*Λ*_M_ (*Λ*_M_ is the period of the interference fringes) and Δ*ω* = *ω*_p2_ *–* *ω*_p1_ is the frequency difference between the two pump laser lines. Projecting this interference pattern on the designed nonlinear metasurface imprints a travelling-wave phase profile, Δ*φ*cos(Δ*ωt−k*_M_*x*), onto the reflected wave. Together with the spatial phase modulation *φ* = *k*_s_*x* created by the distribution of nanoantennas on the metasurface, we obtained a space–time modulation of the form described by Eq. (1). Therefore, the incident wave acquires the energy and momentum shifts when reflected by the metasurface. This process shares some similarities with conventional four-wave mixing in a homogeneous nonlinear material^40^. However, there are subtle but essential differences between them. In our case, we used the meta-atoms to tailor locally both the linear and nonlinear responses—collectively, the metasurface exhibits large temporal phase wobbling under a driving field in addition to a static phase shift, which cannot be achieved using a natural material.

The experimental setup for creating the temporal modulation is illustrated in Fig. 3b and described in the Methods section. In our experiment, the centre wavelength difference was ~6 nm, and the frequency difference was ~2.8 THz (Fig. 3c). In addition, *k*_M_ was adjusted by changing the angle between the two pump beams impinging onto the metasurfaces. Fourier transform analysis of the interference pattern shows a *k*_M_ equal to 0.54*k*_probe_ (Fig. 3d), where *k*_probe_ is the length of the free-space wavevector of the probe beam.

**Fig. 4:**
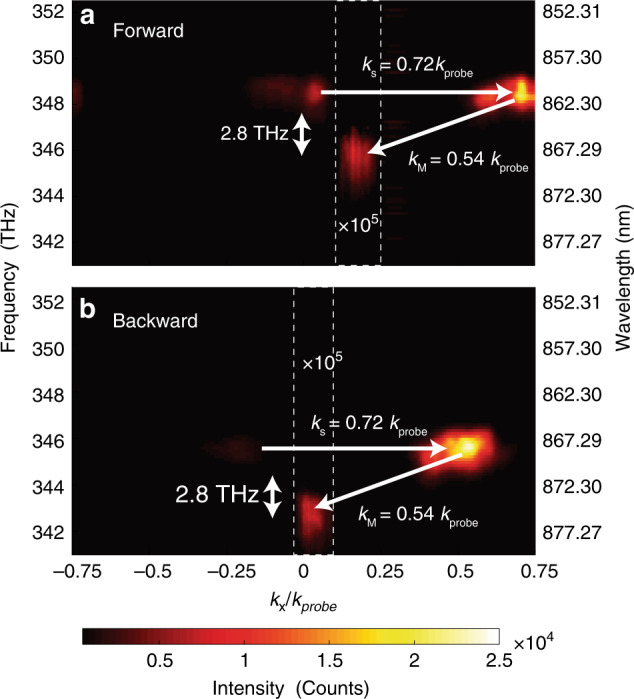
Experimental demonstration of nonreciprocal light reflection on the space–time phase modulated metasurface. **a** Under dynamic modulation with ∆*f* = 2.8 THz and *k*_M_ = 0.54*k*_probe_, the energy-momentum diagram of the normal-incident probe beam (*f* = 348.6 THz) on the metasurface (*k*_s_ = 0.72*k*_probe_) shows a downward converted signal at *f* = 345.8 THz and *k*_x_ = 0.18*k*_probe_. **b** In the backward case, a probe beam with the same frequency as that of the previous signal (*f* = 345.8 THz) but in the opposite direction (*k*_x_ = −0.18*k*_probe_) was sent onto the metasurface. The energy-momentum diagram shows a further down-shifted signal at *f* = 342.0 THz exiting in the normal direction (*k*_x_ = 0). In both cases, the converted signals are magnified by 10^5^ for better illustration (the magnified regions are enclosed by the dashed white boxes). These results perfectly match our theoretical prediction depicted in Fig. 1d, e. In the regions of interest (where all possible photonic transitions exist), finer scanning steps and long spectrometer integration time were used to ensure the detection of converted signals. In the regions with possible upper sidebands or higher-momentum sidebands, no signal was detected even with magnification. Therefore, here, only the regions where the first-order conversions occur are magnified.

To demonstrate the nonreciprocal light propagation, the metasurface with *k*_s_ *=* 0.72*k*_probe_ was imprinted by the interference pattern at a peak pump intensity of ~1 GW/cm^2^, which generated a dynamic phase modulation with ∆*f* = 2.8 THz and *k*_M_ = 0.54*k*_probe_. For the forward propagation experiment, the 860 nm probe light hit the metasurface at normal incidence and resulted in a reflected wave with a shift in the energy-momentum space. This shift was captured by collecting the reflected spectra with a fibre aperture scanning spatially on the Fourier plane (along the *k*_x_ direction) of the focusing lens before the metasurface. Figure 4a displays a static diffraction at 348.6 THz and 0.72*k*_probe_ determined by *k*_s_ and a down-shifted signal at 345.8 THz and 0.18*k*_probe_ produced by the spatiotemporal-modulation-induced ∆*f*, *k*_M_, and *k*_s_. The zeroth-order reflection can also be detected, as the diffraction efficiency dropped due to edge effects and slight polarization misalignment. The aspheric lens used to focus the pump and probe beams and to collect converted signals has an effective NA of 0.76; *k*_x_/*k*_probe_ is therefore bounded by the limited collection angle. On the other hand, the accumulated *k*_x_/*k*_probe_ ~1.26 of the upward transition is greater than unity. Therefore, it is evanescent and cannot carry energy away from the metasurface. For the backward propagation experiment, we sent a probe beam (*f* = 345.8 THz and *k*_x_ = −0.18*k*_probe_) with the same frequency as that of the previous down-converted signal but in the opposite direction onto the metasurface. We observed another downward transition at 343.0 THz exit along the normal direction (Fig. 4b). Similarly, the upward transition is nonexistent since its accumulative *k*_x_ is greater than *k*_probe_. Therefore, the backward propagating light cannot return to the initial state. We also performed a fine scan over the *ω* − *k*_x_ regions where high-order conversions may exist. However, no converted signals were observed since these processes have much lower efficiency. In addition, we performed a control experiment on amorphous silicon film (thickness ~150 nm) using a similar pump intensity, but no converted signal was detected. We experimentally realized nonreciprocal light reflections on the space–time phase modulated metasurface. The experimental results are also confirmed by our finite-difference time domain (FDTD) simulations (Fig. S3). In addition, we investigated the scattering matrix of our space–time phase modulated metasurface. It is asymmetric and hence is direct evidence that our system is nonreciprocal (see S4 of the Supplementary information). To experimentally show the nonreciprocal operation wavelength range of our metasurface, we conducted additional measurements on the nonreciprocal processes by mapping out the dispersion diagrams (Fig. S11) with a narrowband probe, of which the centre wavelength ranges from 854 to 914 nm. By extracting the conversion efficiencies at the tested wavelengths (Fig. S12), a 3-dB bandwidth (full-width at half-maximum, FWHM) of ~5.77 THz was demonstrated (see S6 of the Supplementary information). It is worth noting that in contrast to the waveguide-based systems, the bandwidth of our space–time metasurface is not constrained by the phase-matching conditions. Our experimentally obtained bandwidth is at least one order of magnitude greater than the largest ones reported (a few hundred gigahertz)^9,14^ on time-dependent nonreciprocal systems.

**Fig. 5:**
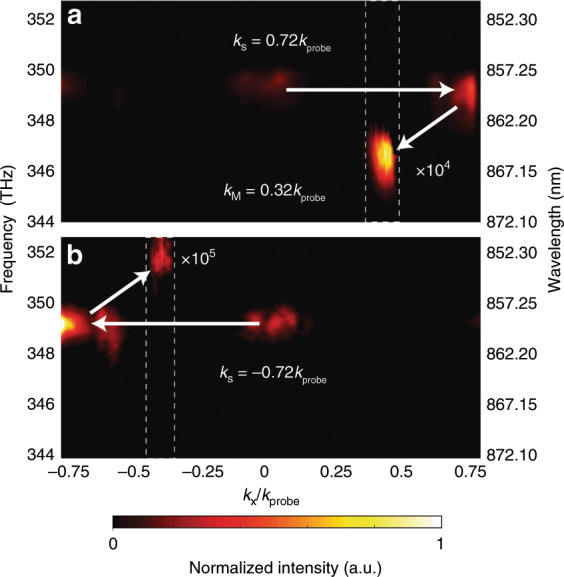
Experimental demonstration of direction selectivity of the photonic transitions. **a** Only downward photonic transition occurs on a metasurface (*k*_s_ = 0.72*k*_probe_) modulated by *k*_M_ = 0.32*k*_probe_ and ∆*f* = 2.8 THz. The converted signal is magnified by 10^4^ for better illustration. **b** In contrast, with the same temporal phase modulation on a metasurface with *k*_s_ = −0.72*k*_probe_, only upward photonic transition occurs. The converted signal is magnified by 10^5^ for better illustration (the magnified regions are enclosed by the dashed white boxes).

Using our space–time metasurface, we are able to independently control the static phase gradient *k*_s_, the dynamic spatial frequency *k*_M_, and the temporal modulation frequency Δ*ω*, which provides unpreceded flexibility in manipulating the photonic transitions. As shown in Fig. 5, by changing *k*_s_, we can selectively enable downward or upward transitions. Additionally, we demonstrated nonreciprocal reflections with arbitrary transverse momenta by tuning both *k*_s_ and *k*_M_ along the metasurfaces (Fig. S10). Moreover, we showed that the modulation frequency can be changed by adjusting the frequency splitting between the two pumps (Fig. S9a). It is worth noting that this level of controllability has not been achieved in previous time-variant nonreciprocal systems^9–16,41^.

## Discussion

We experimentally demonstrated nonreciprocal light reflection based on the space–time modulated nonlinear metasurface. The heterodyne interference created by frequency-shifted pump beams provides robust and controllable spatiotemporal modulation, of which ∆*ω* and *k*_M_ can be readily tuned as desired. It is worth noting that more complex two-dimensional spatiotemporal modulation can be constructed from the heterodyne interference of three or more pump beams. The spatiotemporal phase modulation greatly expands the functions of conventional static phase gradient metasurfaces, providing an additional degree of freedom for manipulating the temporal properties of light and achieving nonreciprocal light propagation. Particularly, we achieved in our experiment a 2.8-THz modulation frequency, a huge step towards optical frequencies, and an ~5.77-THz 3-dB bandwidth, which is orders of magnitude greater than that of current time-variant nonreciprocal systems to the best of our knowledge.

Although travelling-wave-modulation-induced nonreciprocity has already been explored in theory and demonstrated in waveguide systems, such as optical fibre and silicon waveguides, experimental realizations in free space optical systems remain scarce due to the lack of fast, efficient dynamic modulation techniques. Previous demonstrations rely on long interaction lengths (centimetre to metre scale) to amplify the weak dynamic modulation, which makes it difficult to realize in a free space optical system. However, through the use of nonlinear optical metasurfaces to greatly enhance the dynamic modulation strength, optical nonreciprocity is achieved at a subwavelength-scale interaction length on metasurfaces, making this approach potentially compatible with integrated nanophotonic and quantum optical systems. Moreover, by using dynamic phase modulation of a metasurface working in free space, our operation bandwidth is not limited by the group velocity mismatch among guided modes^10,14^. Instead, it is the resonant linewidth of the metasurface that determines the nonreciprocal bandwidth, which is approximately in the range of tens of terahertz.

The conversion efficiency, which is defined as *P*_signal_/*P*_probe_ and is proportional to *J*_1_(Δ*φ*)^2^, is on the order of 2.5 × 10^−4^ in our experiments due to limited experimental conditions. The temporal mismatch (Fig. S6 a) between the pump (~400 fs) and probe (~2 ps) beams limits the effective interaction time between the probe and the dynamic modulation. Due to the poor spatial profile of the probe beam and lens aberration, only ~20% of the probe beam overlaps with the pump beams, resulting in a reduced effective interaction area. In addition, due to the high repetition rate of our laser system (80 MHz), we need to maintain a low pump intensity to avoid significant thermal effects (Fig. S14).

The conversion efficiency of our space–time metasurface can be improved in the following aspects. We find that the conversion efficiency increases super-linearly with the pump intensity in our system (Fig. S14). Using pump light with a low repetition rate and optimized spatiotemporal overlap, we will be able to achieve a higher conversion efficiency. In addition, highly nonlinear materials, e.g., indium tin oxide (ITO), which has large third-order nonlinearity in its epsilon-near-zero region^42,43^, can be used to greatly increase the dynamic phase modulation depth. Furthermore, an optimized nanoantenna design, e.g., a design involving doubly resonant nanoantennas, will improve the temporal phase modulation depth and relax the pump power requirement. In addition, by stacking a pair of space–time phase modulated metasurfaces to form a cavity or integrating metasurface with resonator structures, i.e., micro-ring resonators, the effective interaction length/time will be tremendously increased without sacrificing the device footprint, leading to enhanced nonreciprocal mode conversion efficiency. (A detailed quantitative analysis is included in S7 of the Supplementary information.)

In conclusion, we have demonstrated nonreciprocal light reflection on an ultrafast space–time phase modulated metasurface. This approach exhibits excellent flexibility in controlling optical mode conversions. Moreover, the dynamic system features broken time reversal symmetry compatible with topological insulators, which inspires a new route for isolation using topologically protected edge states^44^ that are immune to disorders. We believe that it will provide a new platform for exploring interesting physics in time-dependent material properties and will open a new paradigm in the development of scalable and integratable nonreciprocal devices.

## Materials and methods

### Sample fabrication

A 200-nm layer of silver was deposited onto a silicon substrate with a 5-nm Ge adhesion layer by electron-beam (e-beam) physical vapour deposition (SEMICORE E-Gun Thermal Evaporator). A 50-nm SiO_2_ dielectric spacer layer and 150-nm amorphous silicon layer were then grown by plasma enhanced chemical vapour deposition (PECVD). The metasurface nanoantennas were created using a sequential process of e-beam lithography (EBL), lift-off of a chromium mask, and inductively coupled plasma-reactive ion etching (ICP-RIE). In the EBL process, we employed a 1:1 diluted ZEP 520A e-beam resist to achieve high resolution. The total pattern size was 200 × 200 μm^2^, written using a Vistec 5200 100 kV e-beam writer. A chlorine-based plasma RIE recipe involving Cl_2_ and Ar gas was used to etch amorphous Si, creating the nanoantennas. The sample was finally immersed in a chrome etchant to remove the chrome mask.

### Simulation method

We developed a two-step full-wave model in a commercial finite element method solver (COMSOL Multiphysics) to obtain the nonlinear Kerr-effect-induced phase shift change (Δ*φ*). The first step iteratively calculates the pump-beam-modified effective permittivity of the structure. The second step simulates a weak probe beam incident onto the structure with the calculated effective permittivity in the first step. By varying the geometrical parameters of the nanoantennas, we obtained the corresponding phase shift changes.

We also performed time-domain simulations using the FDTD to study the spatiotemporal-phase-modulation-induced nonreciprocal photonic transitions. We updated the fields in each time step, taking into account the temporal changes in permittivity. By varying the spatiotemporal modulation parameters Δ*φ*, *k*_s_, *k*_m_, and Δ*ω*, we simulated different conditions corresponding to the experimental demonstrations.

Please refer to section S1 of the Supplementary information for more detailed information on the simulation method.

### Nonreciprocal reflection experiment

To perform measurements of nonreciprocal reflection, the experimental setup shown in Fig. 3b was used. To create two pump beams with a frequency separation for the travelling-wave modulation, the output of a Ti:Sapphire pulsed laser (140-fs pulse width, 80-MHz repetition rate) at *λ* *=* 800 nm was dispersed by a transmission grating and then spatially separated by a customized split mirror with a variable-size block attached in the centre to tune the frequency separation. The spatially separated beams were then reflected back through the grating and recombined into two pump beams with shifted central frequencies. By adjusting delay line 1, the temporal delay between the two pump beams can be tuned. To create the probe beam, the other small portion of the Ti:Sapphire pulsed laser radiation was sent to a nonlinear photonic crystal fibre (PCF) to generate a supercontinuum. Probe light with a 860-nm wavelength was selected using a monochromator. By adjusting delay line 2, the probe can be synchronized with the pumps. An aspheric lens with an effective NA of 0.76 focused the three beams onto the metasurface, which was mounted on a three-dimensional (3D) translation stage. Due to aberration of the aspheric lens, the focal spot of the pump beams was an ellipse with major and minor axis lengths of 50 μm and 45 μm, respectively. The reflected signal was directed to a fibre coupler by a D-shaped pickup mirror, of which the position was adjusted by a linear translation stage to collect the *k*-space information of the output signal.

## Supplementary information

Supplementary Informantion

## References

[1] Caloz C (2018). Electromagnetic nonreciprocity. Phys. Rev. Appl..

[2] Bharadia D, McMilin E, Katti S (2013). Full duplex radios. ACM Sigcomm Comput. Commun. Rev..

[3] Bi L (2011). On-chip optical isolation in monolithically integrated non-reciprocal optical resonators. Nat. Photonics.

[4] Fan L (2012). An all-silicon passive optical diode. Science.

[5] Bender N (2013). Observation of asymmetric transport in structures with active nonlinearities. Phys. Rev. Lett..

[6] Chang L (2014). Parity-time symmetry and variable optical isolation in active-passive-coupled microresonators. Nat. Photonics.

[7] Shi Y, Yu ZF, Fan SH (2015). Limitations of nonlinear optical isolators due to dynamic reciprocity. Nat. Photonics.

[8] Sounas DL, Alù A (2017). Non-reciprocal photonics based on time modulation. Nat. Photonics.

[9] Lira H (2012). Electrically driven nonreciprocity induced by interband photonic transition on a silicon chip. Phys. Rev. Lett..

[10] Correas-Serrano D (2016). Nonreciprocal graphene devices and antennas based on spatiotemporal modulation. IEEE Antennas Wirel. Propag. Lett..

[11] Kang MS, Butsch A, Russell PSJ (2011). Reconfigurable light-driven opto-acoustic isolators in photonic crystal fibre. Nat. Photonics.

[12] Kim JH (2015). Non-reciprocal Brillouin scattering induced transparency. Nat. Phys..

[13] Sohn DB, Kim S, Bahl G (2018). Time-reversal symmetry breaking with acoustic pumping of nanophotonic circuits. Nat. Photonics.

[14] Kittlaus EA (2018). Non-reciprocal interband Brillouin modulation. Nat. Photonics.

[15] Shen Z (2016). Experimental realization of optomechanically induced non-reciprocity. Nat. Photonics.

[16] Ruesink F (2016). Nonreciprocity and magnetic-free isolation based on optomechanical interactions. Nat. Commun..

[17] Yu NF, Capasso F (2014). Flat optics with designer metasurfaces. Nat. Mater..

[18] Ni XJ (2015). An ultrathin invisibility skin cloak for visible light. Science.

[19] Aieta F (2015). Applied optics. Multiwavelength achromatic metasurfaces by dispersive phase compensation. Science.

[20] Chen WT (2018). A broadband achromatic metalens for focusing and imaging in the visible. Nat. Nanotechnol..

[21] Wang SM (2018). A broadband achromatic metalens in the visible. Nat. Nanotechnol..

[22] Ni XJ, Kildishev AV, Shalaev VM (2013). Metasurface holograms for visible light. Nat. Commun..

[23] Zheng GX (2015). Metasurface holograms reaching 80% efficiency. Nat. Nanotechnol..

[24] Lee J (2014). Giant nonlinear response from plasmonic metasurfaces coupled to intersubband transitions. Nature.

[25] Li GX (2015). Continuous control of the nonlinearity phase for harmonic generations. Nat. Mater..

[26] Shaltout A (2014). Optically active metasurface with non-chiral plasmonic nanoantennas. Nano Lett..

[27] Yin XB (2013). Photonic spin hall effect at metasurfaces. Science.

[28] Shaltout AM, Shalaev VM, Brongersma ML (2019). Spatiotemporal light control with active metasurfaces. Science.

[29] Hadad Y, Sounas DL, Alu A (2015). Space-time gradient metasurfaces. Phys. Rev. B.

[30] Shi Y, Fan SH (2016). Dynamic non-reciprocal meta-surfaces with arbitrary phase reconfigurability based on photonic transition in meta-atoms. Appl. Phys. Lett..

[31] Shaltout A, Kildishev A, Shalaev V (2015). Time-varying metasurfaces and Lorentz non-reciprocity. Optical Mater. Express.

[32] Zhang L (2018). Space-time-coding digital metasurfaces. Nat. Commun..

[33] Dai JY (2018). Independent control of harmonic amplitudes and phases via a time-domain digital coding metasurface. Light Sci. Appl..

[34] Liu MK (2018). Huygens’ metadevices for parametric waves. Phys. Rev. X.

[35] Zhang L (2019). Breaking reciprocity with space‐time‐coding digital metasurfaces. Adv. Mater..

[36] Zang JW (2019). Nonreciprocal wavefront engineering with time-modulated gradient metasurfaces. Phys. Rev. Appl..

[37] Eichler, H. J., Günter, P. & Pohl, D. W. *Laser-Induced Dynamic Gratings* (Springer, Berlin, Heidelberg, 2013).

[38] Yu ZF, Fan SH (2009). Complete optical isolation created by indirect interband photonic transitions. Nat. Photonics.

[39] Odoulov S (2015). Interference and holography with femtosecond laser pulses of different colours. Nat. Commun..

[40] Shen Y (1986). Basic considerations of four-wave mixing and dynamic gratings. IEEE J. Quantum Electron..

[41] Fang KJ (2017). Generalized non-reciprocity in an optomechanical circuit via synthetic magnetism and reservoir engineering. Nat. Phys..

[42] Alam MZ, De Leon I, Boyd RW (2016). Large optical nonlinearity of indium tin oxide in its epsilon-near-zero region. Science.

[43] Alam MZ (2018). Large optical nonlinearity of nanoantennas coupled to an epsilon-near-zero material. Nat. Photonics.

[44] Hasan MZ, Kane CL (2010). *Colloquium*: topological insulators. Rev. Mod. Phys..

