# Supplementary material for: Nonreciprocal metasurface with space–time phase modulation
Source: Light Sci Appl. 2019 Dec 18;8:123. doi: 10.1038/s41377-019-0225-z (PMC6920367; doi:10.1038/s41377-019-0225-z)
Supplement: Supplementary file 1 — Supplementary Informantion [file 41377_2019_225_MOESM1_ESM.pdf]

Supplementary Information for

Nonreciprocal Metasurface with Space-Time Phase Modulation

Xuexue Guo, Yimin Ding, Yao Duan, and Xingjie Ni*

Department of Electrical Engineering, Pennsylvania State University, University Park, PA 16802, USA.

*Correspondence to: xingjie@psu.edu

Contents

S1. Simulation methods 2

S1.1 Design of the spatiotemporal phase modulated metasurface 2

S1.2. Finite-difference time-domain (FDTD) simulations of the dynamic phase modulation induced nonreciprocal photonic conversions 4

S2. Optical setup for measuring static properties of the metasurface 10

S3. Pump-probe study of the ultrafast nonlinearity of the metasurface 11

S4. Scattering matrix of the space-time phase modulated metasurface 12

S5. Tunable dynamic modulation 20

S6. Analysis of the operational bandwidth of space-time metasurfaces 24

S7. Analysis of the conversion efficiency and optimization methods 27

References 31

S1. Simulation methods

S1.1 Design of the spatiotemporal phase modulated metasurface

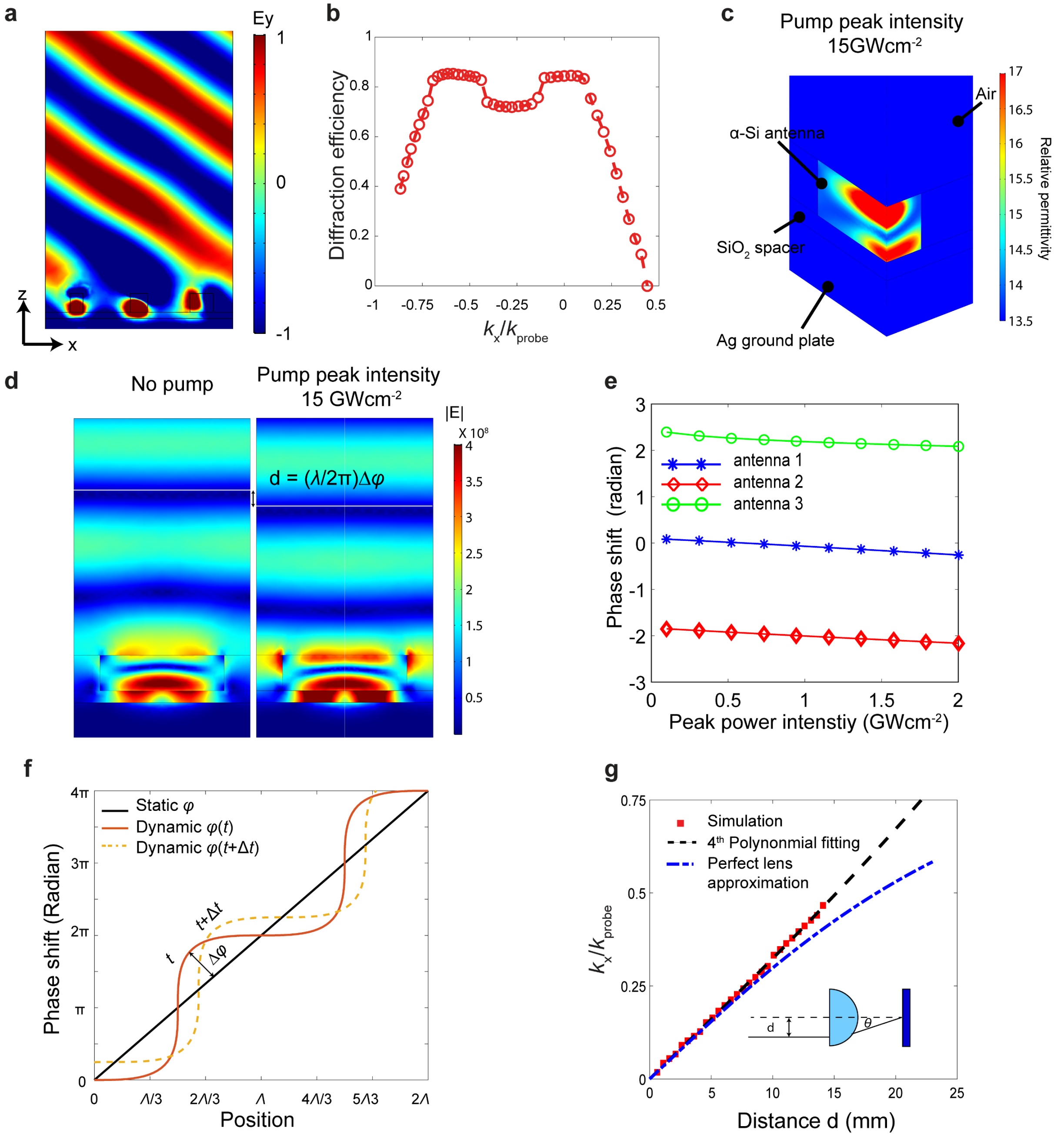

Fig. S1. Simulation of the nonreciprocal metasurfaces. (**a**) A pseudo-color plot of the reflected electric field distribution from a designed metasurface with normal incidence. (**b**) Diffraction efficiency of the metasurface with different incident angles (i.e. different wavevector along the x axis). (**c**) Simulation of the pump (800 nm) induced permittivity change inside a nanoantenna. A

quarter of the antenna is displayed for a better illustration of the distribution of relative permittivity. (**d**) Electrical field distribution of the probe beam (860 nm) reflected from the arrayed nanoantennas (left panel) without and (right panel) with a pump beam, respectively. The abrupt phase shift of the nanoantenna is changed by $\Delta\varphi = 2\pi d/\lambda$, where d is the shift in the wavefront of the reflected light. (**e**) The simulated abrupt phase shifts of the three constituent nanoantennas vs. peak power intensity of the pump. (**f**) Reflection phase shift distribution across the modulated metasurface, with black solid line representing the static phase distribution, red solid line representing the spatiotemporally modulated phase at time instant t and yellow dashed line representing the phase at $t+\Delta t$. (**g**) The relation between k_x/k_{probe} and the distance of pickup mirror relative to the lens axis derived from raytracing (Zemax) simulations (red squares) and perfect lens approximation (blue dashed line).

Numerical simulations were carried out using a commercially available finite element method (FEM) solver package – COMSOL Multiphysics – with periodic boundary conditions for a single building block. Third-order finite elements and at least 10 mesh steps per wavelength were used to ensure the accuracy of the calculated results. The experimentally obtained optical constants of silver and amorphous silicon were used to model the back reflector and the nanoantennas. The refractive index of the spacer layer (SiO_2) was chosen to be 1.45. The scattered field formulation was used to calculate the reflected light at λ = 860 nm. By sweeping the size of nanoantennas (l_x and l_y), we mapped out the static phase shifts which cover over 2π range, and selected three nanoantennas to construct a unit supercell (Fig. 2b). Fig. S1a illustrates the simulated electric field distribution of a supercell at normal incidence (λ = 860 nm), showing a smoothly slanted wavefront. We also simulated the diffraction efficiency under different incident angles which are transformed into wavevectors along the x axis. As shown in Fig. S1b, the metasurface keeps a high diffraction efficiency above 84% near normal incidence (within +/− 10 degrees).

The nonlinear simulation takes two major steps in a customized model using COMSOL Multiphysics: (1) We simulated the spatially dependent permittivity change using an iterative scheme – We first calculated the field distribution in the computational domain with the pump beam incidence, then updated the permittivity in the Kerr medium ($n_2 = 5 \times 10^{-13}$ cm^2W^{-1} [1]) with the calculated inhomogeneous field, and after that we calculated the field again with modified

permittivity. We iterated over the steps above until the change of the field distribution was within a predefined tolerance. As shown in Fig. S1c, pumped at a peak intensity of 15 $\mathrm{GWcm^{-2}}$ by 800 nm light, the nanoantenna shows a significant permittivity change thanks to the resonance-enhanced local field and large nonlinear Kerr index of amorphous silicon. (2) We then simulated the structure with the pump-induced permittivity changes with a probe light incidence. With the nonlinear simulation, we obtained the reflected field distribution of an 860 nm probe beam upon incidence on an arrayed nanoantennas whose permittivity has been modified by the pump beam. An observable wavefront shift, $d = (\lambda/2\pi)\Delta\varphi$, related to the phase shift change is shown in Fig. S1d. With increasing pumping intensity, the phase shifts induced by the three elemental nanoantennas show a uniform change (Fig. S1e), which is important for keeping a well-aligned dynamic phase modulation across the whole antennas array (Fig. S1f).

In order to map the position of the pickup mirror relative to the tangential momentum k_x of the reflected beam, we used a raytracing software package (Zemax) to simulate the ray trajectory of light focused by the aspheric lens used in our experiments. By varying the distance between the incident beam and the axis of the lens, different focusing angle θ after the lens are obtained and transformed into k_x by $k_x = \sin(\theta) k_{\mathrm{probe}}$. As shown in Fig. S1g, this method has a better precision than the calculation made by treating the aspheric lens as a perfect thin lens, especially for off-axis rays.

S1.2. Finite-difference time-domain (FDTD) simulations of the dynamic phase modulation induced nonreciprocal photonic conversions

To verify the theoretical predictions of equation (2), we performed FDTD simulations of the dynamically phase modulated metasurface which is simplified as a dipole array. A phase modulation of the form $k_s x_n + \Delta\varphi \cos(\Delta\omega t - k_M x_n)$ was assigned to each dipole, where x_n is the x coordinate of the dipole n. This simplification provides insight into the nonreciprocal photonic transitions from space-time phase modulation and allows us to simulate a large area of the metasurface with a reasonable amount of computational resources.

In the first set of simulations, we simulated the case where both up and down-conversions are allowed. The model consists of three supercells of metasurfaces with a period of 1200 nm (k_s =

$0.72k_{\text{probe}}$, $\lambda_{\text{probe}} = 860$ nm), and three nanoantennas (represented by three dipoles) spanning a 2π phase shift range are included in each supercell. As shown in Fig. S2a, when we sent in a normally incident plane wave at $\lambda = 860$ nm, in consistency with the conversions shown in the dispersion diagram, the reflected plane waves are composed of three frequency/wavevector components (1) The static diffraction is deflected to an angle of $\theta = \sin^{-1}\left(k_s / k_{probe} \right) = 45.78°$, (2) the up-conversion ($\lambda = 853$ nm) is deflected to an angle of $\theta = \sin^{-1}\left((k_s - k_M) / k_{probe} \right) = 72.85°$, and (3) the down-conversion signal ($\lambda = 867$ nm) is deflected to an angle of $\theta = \sin^{-1}\left((k_s + k_M) / k_{probe} \right) = 28.54°$.

In the backward propagation scenario, we sent back the two converted signals from the forward propagation case. Here, as we had two input fields, the field distribution of the outgoing waves would be a superposition of multiple plane waves. In order to clearly visualize the outgoing waves, we separated the converted waves arisen from the two backward inputs and presented their electric field distributions at modulated wavelengths in Fig. S2b and c, respectively. The backward propagation of the down-converted signal ($\lambda = 867$ nm) results in an upward converted wave at $\lambda = 860$ nm with a reflection angle of $\theta = \sin^{-1}\left(-(k_s - k_M) + k_M + k_s / k_{probe} \right) = -28.54°$ and a downward converted wave of $\lambda = 874$ nm with a reflection angle of $\theta = \sin^{-1}\left(-(k_s - k_M) - k_M + k_s / k_{probe} \right) = 0°$ (Fig. S2b). The backward propagation of the up-converted signal ($\lambda = 853$ nm) results in a down-conversion at $\lambda = 860$ nm with a reflection angle of $\theta = \sin^{-1}\left(-(k_s + k_M) - k_M + k_s / k_{probe} \right) = 28.54°$ and an up-conversion of $\lambda = 866$ nm with a reflection angle of $\theta = \sin^{-1}\left(-(k_s + k_M) + k_M + k_s / k_{probe} \right) = 0°$ (Fig. S2c). None of the back-reflections return to the state of the original forward incidence, which proves that the spatiotemporal phase modulated metasurface is nonreciprocal.

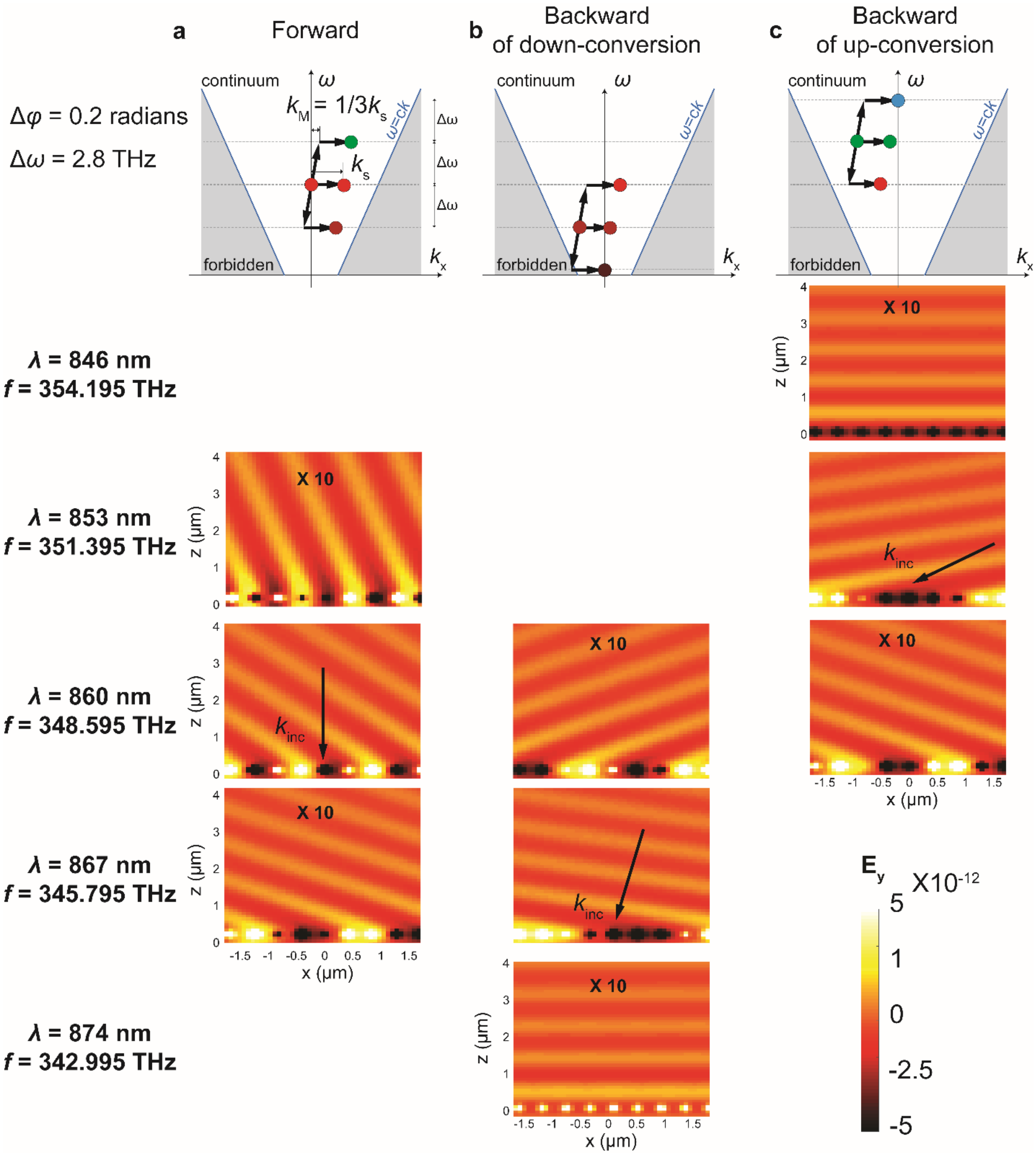

Fig. S2. **FDTD simulation of the space-time phase modulated metasurface with both upward and downward photonic transitions using a simplified dipole array.** Here we used $k_s = 0.72k_{probe}$, $k_M = 1/3\ k_s = 0.24k_{probe}$, $\Delta\omega = 2.8$ THz, and $\Delta\varphi = 0.2$ radians to allow both up and down-conversions. (**a**) The y component of the electric field (E_y) distributions of the three outgoing waves from a normal incident plane wave at $\lambda = 860$ nm. From top to bottom, the field plots show the

first-order up-conversion, the static diffraction, and the first-order down-conversion, respectively. The k_x-ω dispersion diagram shows the conversions which fulfil the momentum and energy conservations. (**b**) The incident wave (indicated by the black arrow) is switched to a plane-wave that is the backward of the down-conversion from (**a**). E_y of the outgoing waves arisen from the first-order conversions and the static diffraction are shown and their frequencies (i.e. energy) and k_x (i.e. momentum) agree with the transitions shown in the k_x-ω dispersion diagram. None of the outgoing waves follow the same path of the normal incidence of (**a**). (**c**) In the backward reflection of the up-conversion from (**a**) (indicated by the black arrow), three outgoing waves indicated by the k_x-ω dispersion diagram are shown to have different frequencies or reflection angles from the normal incidence in (**a**). In all E_y distribution plots, the field strength of the static diffraction one order of magnitude stronger than the converted signals. This is consistent with the relation shown in equation (2) in the main text, where the static diffraction efficiency is about $|J_0(0.2)|^2 = 0.98$ and the first order conversion efficiency is about $|J_1(0.2)|^2 = 0.0099$.

In the second set of simulations, we studied the case where only one direction of transition is allowed. Fig. S3 shows the simulation results of dipoles array with $k_s = 0.78k_{probe}$, $k_M = 2/3\ k_s = 0.52k_{probe}$, $\Delta\omega = 2.8$ THz, and $\Delta\varphi = 0.2$ radians. In the forward propagation, the normal incidence ($\lambda = 860$ nm) is converted to two outgoing waves: the static diffraction and the first-order down-conversion. The up-conversion corresponds to a non-propagating mode that cannot radiate into free space. As shown in the field plot, this evanescent mode cannot carry energy away from the metasurface and no propagating field can be observed in free space. In the backward case, the incident wave is the backward of the down-conversion (Fig. S3a). Only the down-conversion at $\lambda = 874$ nm can be observed at the normal direction. Similarly, the up-conversion results in a non-propagating mode that cannot carry energy away.

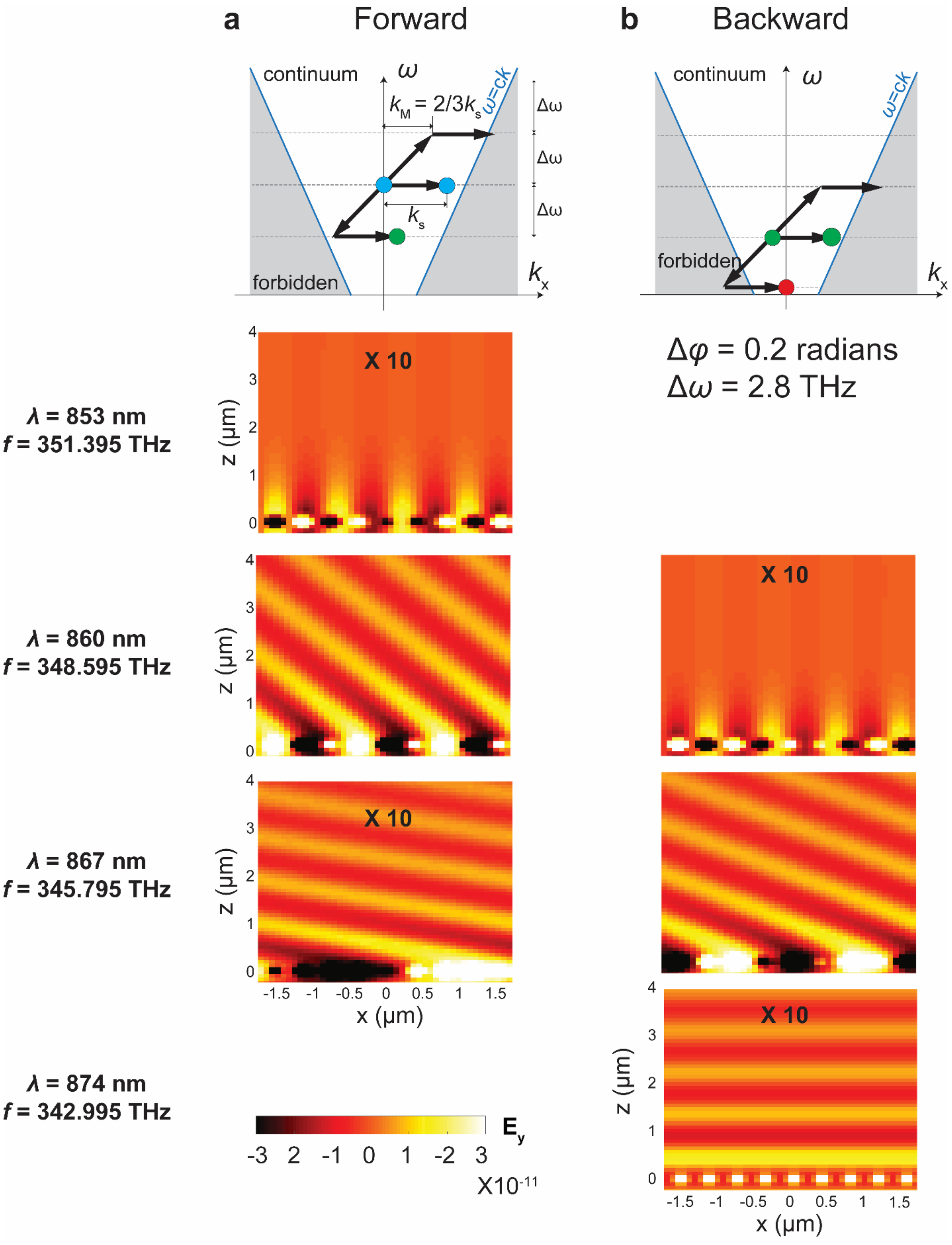

Fig. S3. FDTD simulation of the space-time phase modulated metasurface with only

downward photonic transitions using a simplified dipole array. Here we used $k_s = 0.78k_{probe}$ and $k_M = 2/3\ k_s = 0.52k_{probe}$ to allow only unidirectional down-conversions. (**a**) With a phase modulation depth $\Delta\varphi = 0.2$ radians, the forward normal incidence at $\lambda = 860$ nm is diffracted to a static diffraction and a first-order down-conversion. E_y plot of the first-order up-conversion shows no propagating wave existing in free space, which is also confirmed by the dispersion diagram and demonstrated in the experiment. (**b**) The incident wave is switched to the backward of the down-conversion shown in (**a**). In response to this incidence, a down-conversion at $\lambda = 874$ nm reflects at the normal angle, while no propagating mode exists at $\lambda = 860$ nm. None of the outgoing waves overlaps with the forward incidence, and the process is nonreciprocal.

We also simulated the ideal case where the static diffraction vanishes under $J_0(\Delta\varphi) = 0$ ($\Delta\varphi = 2.405$ radians). In this case, the electric field strength of the static diffraction is negligible, and we can achieve near unitary conversion efficiency and ultra-high isolation ratio (Fig. S4)

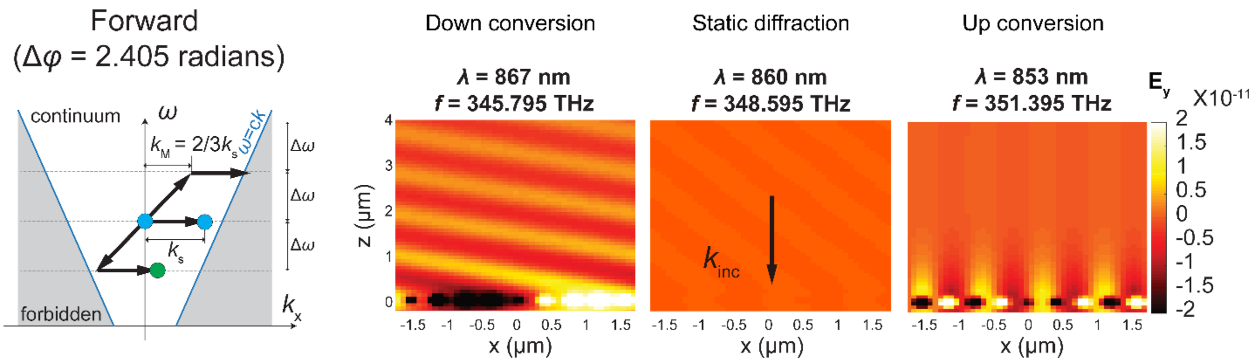

Fig. S4. Complete photonic transitions with ideal temporal phase modulation depth $\Delta\varphi$ = 2.405 radians. The phase modulation parameters are the same as Fig. S3 except that $\Delta\varphi$ is equal to 2.405 radians leading to $J_0(\Delta\varphi) = 0$. The static diffraction of the metasurface is almost completely eliminated.

S2. Optical setup for measuring static properties of the metasurface

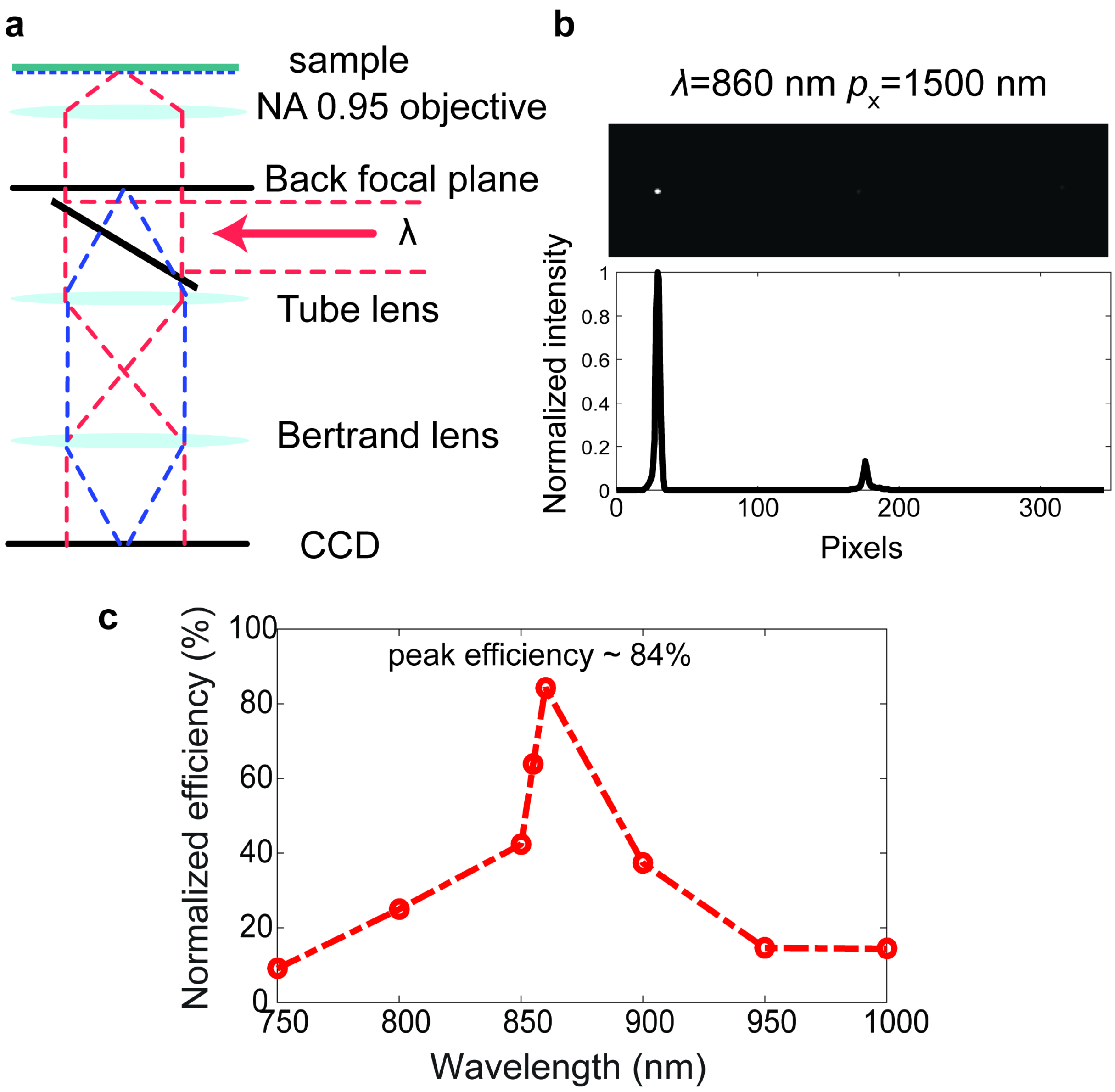

Fig. S5. Characterization of the metasurface. (**a**) A schematic of the *k*-space imaging setup used to measure the reflection angle and static diffraction efficiency of the fabricated metasurface. (**b**) One example of the *k*-space image with λ = 860 nm and p_x = 1500 nm. The bottom panel shows a normalized intensity profile extracted from the *k*-space image. (**c**) Experimentally measured static diffraction efficiencies of the sample with p_x = 1500 nm at different wavelengths.

To characterize the sample's static diffraction properties, we adopted the *k*-space imaging technique based on an inverted microscope (Nikon Eclipse TE 2000U). As shown in Fig. S5a, the red dashed line represents the real space imaging light path, and the blue dashed line traces the *k*-space imaging process. The sample was illuminated by a Ti:Sapphire femtosecond laser through an objective (Nikon Plan Fluor 60X/0.95 NA), and the reflected light was collected by the same objective. To acquire two-dimensional (2D) *k*-space images, the back focal plane of the objective was directly imaged by a Bertrand lens before a charge-coupled device (CCD) camera. Fig. S5b depicts one *k*-space image and its intensity profile, showing a high static diffraction efficiency of the metasurface. Based on the *k*-space measurements, the diffraction efficiency of the metasurface was extracted and calibrated (Fig. S5c). It exhibits a high efficiency of 84% near 860 nm.

S3. Pump-probe study of the ultrafast nonlinearity of the metasurface

We performed the pump-probe experiment by monitoring the down-conversion signal change with different delay time between the probe beam and the pump beams. To study the temporal response of the metasurface, we first characterized the temporal information of the pump and probe beams used in our experiment. We carried out the pumps correlation and probe-pump cross-correlation experiments using the setup shown in Fig. 3b. The top panel in Fig. S6a shows the correlation sum frequency generation (SFG) signal of the two frequency shifted pump beams at different delay by adjusting the delay line 1. The pulse width of the Ti:Sapphire laser is 140 fs. It is stretched to 400 fs after passing through dispersive optical components and split into two frequency-shifted components with half of the original bandwidth. The middle panel is the cross-correlation of the probe and the blue-shifted pump beam measured by monitoring the SFG signal at different delay by adjusting the delay line 2. At around 860 nm, the pulse width of the probe is approximately 2 ps.

Next, the metasurface was pumped by the two frequency-shifted pump beams and probed by a weak beam of 869 nm. We monitored the down-conversion signal (λ = 876 nm) shown in Fig. S6b at different probe-pumps delay by adjusting delay line 2. Due to the limitation of the probe pulse width (~2 ps), our experiment condition cannot obtain the exact response time of the nonlinear Kerr effect of amorphous silicon metasurface. However, the down-conversion signal evolution follows the same envelope of the pump-probe cross correlation, proving that the Kerr nonlinear

response time of the metasurface is at least within picosecond scale and thus ruling out the involvement of thermal effect in the phase modulation. According to the Ref. [2], the nonlinear Kerr effect response time of amorphous silicon wire waveguide is less than 100 fs. Thus our pump-probe experiment confirmed that the dynamic phase changes is originated from the nonlinear Kerr effect and ruled out the involvement of thermal effect which is much slower.

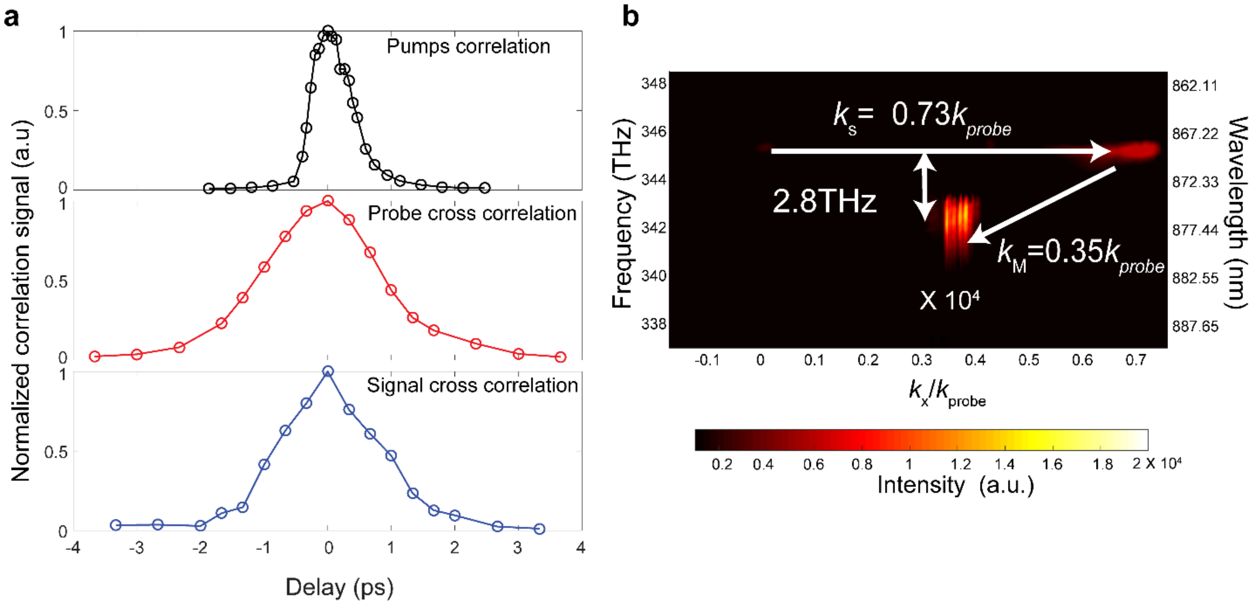

Fig. S6. Pump-probe experiment of the transition signal of metasurface. (**a**) The cross-correlation signal of two frequency-shifted pump beams (top panel), the cross-correlation signal of the probe and the pump (middle panel), and the evolution of down-converted signal (f = 342.4 THz, λ = 876 nm) with different delay of the pumps. (**b**) k-space frequency distribution of the down conversion process of a normal incidence (f = 345.2 THz, λ = 869 nm) upon the metasurface ($k_s = 0.73k_{probe}$) and modulated by $\Delta\omega$ = 2.8 THz and k_M = 0.35 k_{probe}. The down-conversion signal is monitored while adjusting the delay line 2, and the results are plotted in the bottom figure of (**a**). The down-converted signal (f = 342.4 THz, λ = 876 nm, and $k_x = 0.38k_{probe}$) is scaled by 10^4 for better illustration.

S4. Scattering matrix of the space-time phase modulated metasurface

In our design, nonreciprocity is achieved through asymmetric photonic transitions induced by the spatiotemporal phase modulation. In the main text, we demonstrated in theory and experiment that

unidirectional photonic transitions can break the reciprocity. Although the up and down conversions can coexist under certain conditions, e.g. small k_M and small k_s, the nonreciprocal effect arisen from the time-dependent phase modulation of metasurface will not be compromised. We will demonstrate the nonreciprocity by deriving the scattering matrix of the system. As shown in Fig. S7, we considered our metasurface as a four-port system, where s_{1+} is the amplitude of the incident wave (ω_i, k_{ix}), s_{2-} is the amplitude of the static diffraction (ω_i, $k_{ix} + k_s$), s_{3-} is the amplitude of the down-conversion ($\omega_i - \Delta\omega$, $k_{ix} + k_s - k_M$) and s_{4-} is the amplitude of the up-conversion ($\omega_i + \Delta\omega$, $k_{ix} + k_s + k_M$). Table 1 summarizes the frequency and wavevector of each port.

**Port number**	**Input s_+**	**Output s_-**
1	ω_i, k_{ix}, k_{iz}	ω_i, $-k_{ix}$, $-k_{iz}$
2	ω_i, $k_{ix} + k_s$, k_{2z}	ω_i, $-(k_{ix} + k_s)$, $-k_{2z}$
3	$\omega_i - \Delta\omega$, $k_{ix} + k_s - k_M$, k_{3z}	$\omega_i - \Delta\omega$, $-(k_{ix} + k_s - k_M)$, $-k_{3z}$
4	$\omega_i + \Delta\omega$, $k_{ix} + k_s + k_M$, k_{4z}	$\omega_i + \Delta\omega$, $-(k_{ix} + k_s + k_M)$, $-k_{4z}$

Table 1: The frequencies and wavevectors of the waves related to the four ports.

As mentioned in the main text, the reflected light from the metasurface will acquire a dynamic phase and be decomposed into components of different frequencies and wavevectors, as expressed below:

$$\vec{E}_{\mathrm{r}}(\vec{r},t)=\zeta J_0(\Delta\varphi)\vec{E}_{\mathrm{i}}e^{i(\vec{k}_{\mathrm{i}}\vec{r}+k_{\mathrm{s}}x-\omega_{\mathrm{i}}t)}+i\zeta J_1(\Delta\varphi)\vec{E}_{\mathrm{i}}\left\{e^{i\left[(\vec{k}_{\mathrm{i}}\vec{r}+k_{\mathrm{M}}x+k_{\mathrm{s}}x)-(\omega_{\mathrm{i}}+\Delta\omega)t\right]}+e^{i\left[(\vec{k}_{\mathrm{i}}\vec{r}-k_{\mathrm{M}}x+k_{\mathrm{s}}x)-(\omega_{\mathrm{i}}-\Delta\omega)t\right]}\right\} \quad \text{(S1)}$$

where $\zeta = \sqrt{\eta}$ and η is the static diffraction efficiency of the metasurface, $\vec{E}_i$ is the electric field of the incident wave, $\Delta\varphi$ is the amplitude (or modulation depth), k_s is the static phase gradient of the metasurface, $\Delta\omega$ is the dynamic modulation frequency and k_M is the modulation wavevector. High-order modes are not considered here since they are very weak with small phase modulation depth. We also omitted the specular reflection because the metasurface diffraction efficiency is close to unity around the designed wavelength.

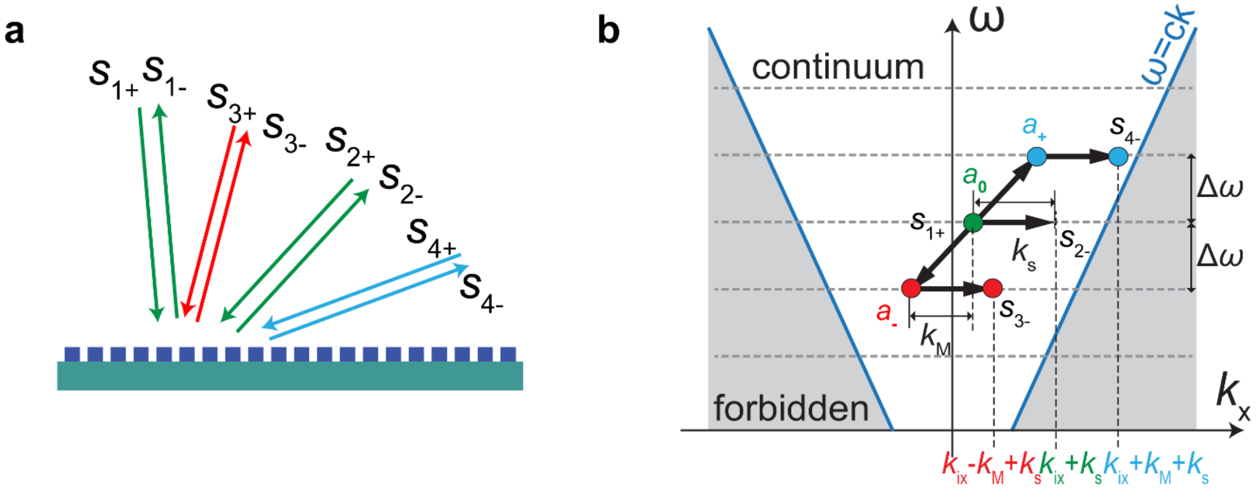

Fig. S7 (**a**) Illustration of a four-port system representing the metasurface, where port 1 is the incident port, port 2 relates to the static diffraction of the metasurface, and port 3 (4) couples to the down (up) converted mode. (**b**) A dispersion diagram depicting the energy/momentum relationships between modes a_0, a_-, and a_+. Mode a_0 (a_- and a_+) will couple out to s_{2-} (s_{3-} and s_{4-}) due to the static phase gradient k_s of the metasurface.

According to equation (S1), we can relate the s_{i-} to s_{i+} by using the following equations:

$$
\begin{aligned}
s_{1-} &= \zeta J_0(\Delta\varphi) e^{ik_s x} s_{2+} \\
s_{2-} &= \zeta J_0(\Delta\varphi) e^{ik_s x} s_{1+} \\
s_{3-} &= i\zeta J_1(\Delta\varphi) e^{i\Delta\omega t} e^{-ik_M x} e^{ik_s x} s_{1+} \\
s_{4-} &= i\zeta J_1(\Delta\varphi) e^{-i\Delta\omega t} e^{ik_M x} e^{ik_s x} s_{1+}
\end{aligned}
\tag{S2}
$$

Therefore, the scattering matrix of the four-port system can be written as:

$$
S = \begin{pmatrix}
0 & \zeta J_0(\Delta\varphi) & 0 & 0 \\
\zeta J_0(\Delta\varphi) & 0 & 0 & 0 \\
i\zeta J_1(\Delta\varphi) e^{i\Delta\omega t} e^{-ik_M x} & 0 & 0 & 0 \\
i\zeta J_1(\Delta\varphi) e^{-i\Delta\omega t} e^{ik_M x} & 0 & 0 & 0
\end{pmatrix}
\tag{S3}
$$

The common factor $e^{ik_s x}$ is omitted since it does not influence the form of the scattering matrix.

It is evident that the scattering matrix is asymmetric ($S_{13} \neq S_{31}$ and $S_{14} \neq S_{41}$) due to the asymmetric photonic transitions arisen from spatiotemporal phase modulation. Thus, the system breaks reciprocity. S_{12} (S_{21}) represents the insertion loss to the nonreciprocal system. Under ideal condition, it could be reduced to zero when $J_0(\Delta\varphi) = 0$ (e.g. $\Delta\varphi = 2.405$ radians).

In order to validate the scattering matrix obtained directly from field calculation, we also studied the dynamic system based on the coupled mode theory [3,4]. Considering the same four-port system shown in Fig. S7b, the incident wave through port 1 will directly excite the resonant mode a_0:

$$a_0\left(t\right)=\tilde{a}_0 e^{-i\omega_0 t} \tag{S4}$$

The dynamic phase modulation with form $\Delta\varphi\cos(\Delta\omega t-k_{\mathrm{M}}x)$ can be translated into effective permittivity modulation of the metasurface, which can be expressed as $\Delta\varepsilon(x,t)=\Delta\varepsilon_{eff}\cos(\Delta\omega t-k_M x)$. The permittivity modulation induces the coupling among modes a_0, a_-, and a_+. Therefore, we can obtain a set of master equations for the dynamics of the three excited modes as shown in Fig. S7b:

$$\begin{aligned}
\dot{a}_0 &= (-i\omega_0-\gamma_0)a_0+i\kappa e^{-i\Delta\omega t}e^{ik_M x}a_- + i\kappa e^{i\Delta\omega t}e^{-ik_M x}a_+ + c_1 s_{1+} \\
\dot{a}_- &= (-i\left(\omega_0-\Delta\omega\right)-\gamma_-)a_- + i\kappa e^{i\Delta\omega t}e^{-ik_M x}a_0 \\
\dot{a}_+ &= (-i\left(\omega_0+\Delta\omega\right)-\gamma_+)a_+ + i\kappa e^{-i\Delta\omega t}e^{ik_M x}a_0
\end{aligned} \tag{S5}$$

where γ represents the decay rate of the modes, c_i is the coupling coefficient between the port i and the mode, κ represents the coupling strength between the modes induced by the permittivity modulation. The outgoing waves are expressed as:

$$\begin{aligned}
s_{1-} &= S_{12}s_{2+} \\
s_{2-} &= c_2 a_0 \\
s_{3-} &= c_3 a_- \\
s_{4-} &= c_4 a_+
\end{aligned} \tag{S6}$$

where port 1 and port 2 are reciprocally connected because they are linked by the static phase gradient of metasurface which conserves reciprocity. Thus, $S_{12}=S_{21}$. We consider that an incident wave ($s_{1+}(t)=\tilde{s}_{1+}e^{-i\omega t}$) is excited at frequency ω. Therefore $a_0\left(t\right)=\tilde{a}_0 e^{-i\omega t}$, $a_-\left(t\right)=\tilde{a}_- e^{-i\omega t}$, and $a_+\left(t\right)=\tilde{a}_+ e^{-i\omega t}$, where $\tilde{a}_0$, $\tilde{a}_-$ and $\tilde{a}_+$ are the modal amplitudes for mode a_0, a_-, and a_+, respectively. By substituting $A_0\left(t\right)=a_0(t), A_-\left(t\right)=a_-(t)e^{-i\Delta\omega t}$, and $A_+\left(t\right)=a_+(t)e^{i\Delta\omega t}$, we obtain the following solutions:

$$
\begin{aligned}
A_0(t) &= \frac{c_1\left(\gamma_- + i\left(\omega_0-\omega\right)\right)\left(\gamma_+ + i\left(\omega_0-\omega\right)\right)}{D} s_{1+}(t) \\
A_-(t) &= \frac{i\kappa e^{-ik_M x} c_1\left(\gamma_+ + i\left(\omega_0-\omega\right)\right)}{D} s_{1+}(t) \\
A_+(t) &= \frac{i\kappa e^{ik_M x} c_1\left(\gamma_- + i\left(\omega_0-\omega\right)\right)}{D} s_{1+}(t)
\end{aligned}
\tag{S7}
$$

where

$$
D = \left(\gamma_0 + i\left(\omega_0-\omega\right)\right)\left(\gamma_- + i\left(\omega_0-\omega\right)\right)\left(\gamma_+ + i\left(\omega_0-\omega\right)\right) + \kappa^2\left(\gamma_- + i\left(\omega_0-\omega\right)\right) + \kappa^2\left(\gamma_+ + i\left(\omega_0-\omega\right)\right)
$$

Therefore, the scattering matrix of the system can be expressed as:

$$
S = \begin{pmatrix}
0 & \dfrac{c_1 c_2\left(\gamma_- + i\left(\omega_0-\omega\right)\right)\left(\gamma_+ + i\left(\omega_0-\omega\right)\right)}{D} & 0 & 0 \\
\dfrac{c_1 c_2\left(\gamma_- + i\left(\omega_0-\omega\right)\right)\left(\gamma_+ + i\left(\omega_0-\omega\right)\right)}{D} & 0 & 0 & 0 \\
\dfrac{i\kappa e^{i\Delta\omega t} e^{-ik_M x} c_1 c_3\left(\gamma_+ + i\left(\omega_0-\omega\right)\right)}{D} & 0 & 0 & 0 \\
\dfrac{i\kappa e^{-i\Delta\omega t} e^{ik_M x} c_1 c_4\left(\gamma_- + i\left(\omega_0-\omega\right)\right)}{D} & 0 & 0 & 0
\end{pmatrix}
\tag{S8}
$$

Because $\Delta\omega$ (~ 2.8 THz × 2π) is much smaller than ω_0 and the bandwidth of the metasurface resonance is much larger than $\Delta\omega$, we can assume $\gamma_0 = \gamma_- = \gamma_+ = \gamma$. In addition, we can also assume $c_1 = c_2 = c_3 = c_4 = c$ because in a lossless system the coupling strength c between a port and a resonant mode is linked with the mode's radiative decay rate $c = \sqrt{2\gamma}$. [5]. Therefore, when the $\omega = \omega_0$, the scattering matrix can be further simplified as

$$
S = \begin{pmatrix}
0 & \dfrac{c^2\left(\gamma^2 + \Delta\omega^2\right)}{D} & 0 & 0 \\
\dfrac{c^2\left(\gamma^2 + \Delta\omega^2\right)}{D} & 0 & 0 & 0 \\
\dfrac{i\kappa c^2 \gamma e^{i\Delta\omega t} e^{-ik_M x}}{D} & 0 & 0 & 0 \\
\dfrac{i\kappa c^2 \gamma e^{-i\Delta\omega t} e^{ik_M x}}{D} & 0 & 0 & 0
\end{pmatrix}
\tag{S9}
$$

where $D=\gamma\left(\gamma^2+\Delta\omega^2\right)+2\kappa^2\gamma$.

By comparing equation (S9) with equation (S3), we found that the scattering matrices derived from two difference methods are of the same form, in which $S_{12}=S_{21}$, $|S_{31}|=|S_{41}|$, $S_{13}=S_{14}=0$, as well as $S_{13}\neq S_{31}$ and $S_{14}\neq S_{41}$. Therefore, both derivations reach the same conclusion – the dynamically phased modulated metasurface possesses an asymmetric scattering matrix and is nonreciprocal.

In order to prove the validity of the scattering matrix we derived above, we experimentally demonstrated the nonreciprocal light reflection when both up and down-conversions are presented. As shown in Fig. S8a, the normal incident light (green) has Stokes (red) and anti-Stokes (blue) shifts, whose frequencies are shifted down and up by $\Delta\omega$, respectively. The reverse of the case above will consist of two paths: (a) the backward propagation of the up-converted signal (blue) and (b) the backward propagation of the down-converted signal (red). First, we consider path (a). The up-converted signal (blue) is sent back towards the metasurface and again has Stokes (green) and anti-Stokes (purple) shifts, where the Stokes (green) reflects at an angle ($\sin\theta=-2\frac{k_M}{k_0}$) different from the normal direction, and the anti-stokes (purple) exits at the same angle of the original forward incidence but with frequency shifted up by $2\Delta\omega$. We can see that not all light returns to the angle same as the original incidence even if we do not consider the shifts in frequency. It is sufficient to conclude that our system is nonreciprocal because the reciprocity of the system requires both the Stokes and anti-Stokes shifts go back to the original incident state. Path (b) will have similar processes, and we will not elaborate here for the sake of conciseness.

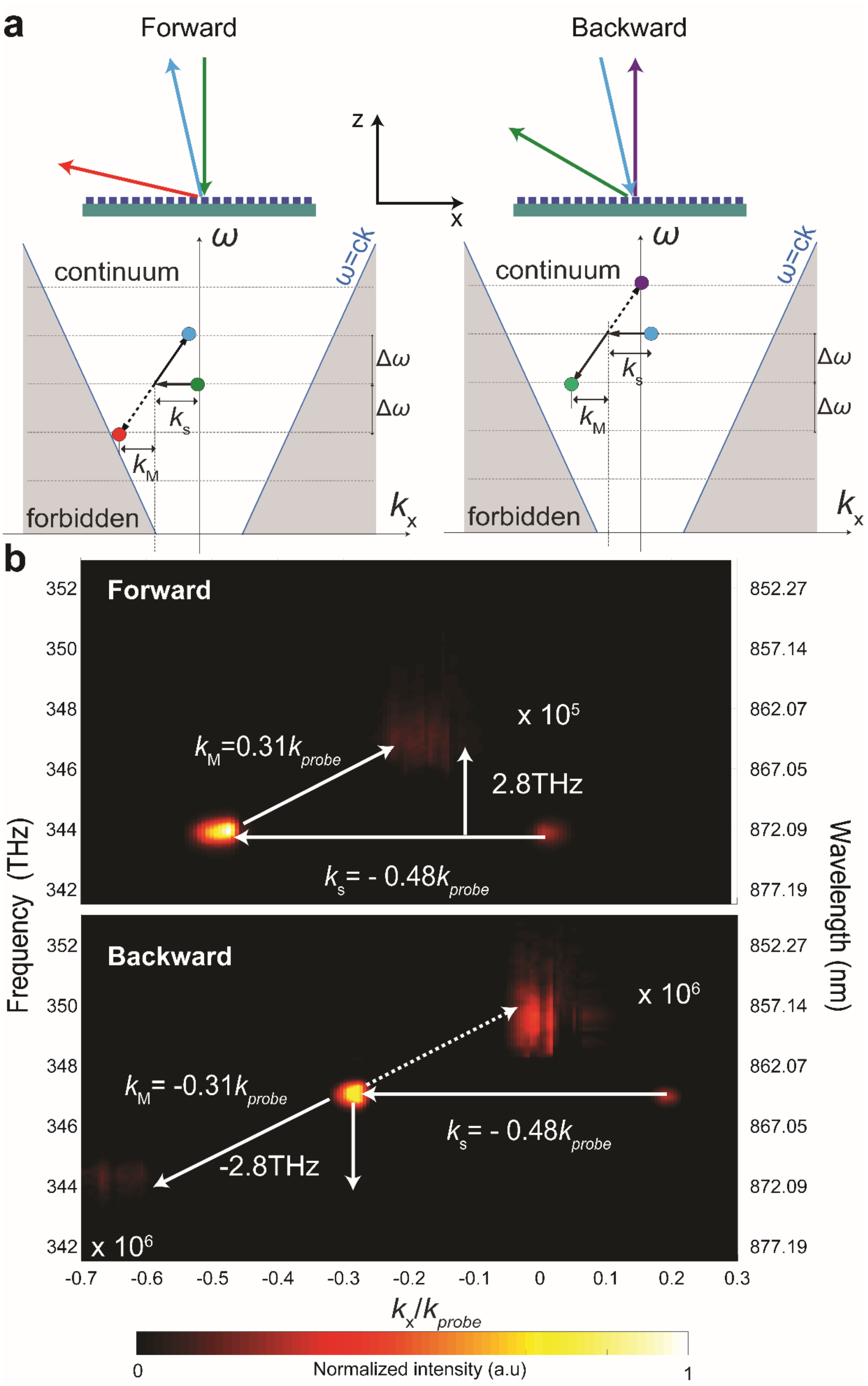
a
Forward
Backward
z
x
ω
continuum
ω=ck
Δω
Δω
k_s
k_M
forbidden
k_x
b
Forward
x 10^5
k_M=0.31k_probe
2.8THz
k_s= - 0.48k_probe
Backward
x 10^6
k_M= -0.31k_probe
k_s= - 0.48k_probe
-2.8THz
x 10^6
Frequency (THz)
Wavelength (nm)
352
350
348
346
344
342
852.27
857.14
862.07
867.05
872.09
877.19
-0.7
-0.6
-0.5
-0.4
-0.3
-0.2
-0.1
0
0.1
0.2
0.3
k_x/k_probe
0
Normalized intensity (a.u)
1

Fig. S8 (**a**) Schematics and dispersion diagrams of nonreciprocal light reflections where both up and down-conversions are present from the dynamically modulated metasurfaces. In the forward case, the normal incidence (green) will be converted into a down-conversion signal (red) and an up-conversion signal (blue) obeying the conservation of both energy and momentum. The backward reflection of the up-conversion signal (blue) will be converted upwards (purple) and downwards (green), where either the frequency or the transversal wavevector of the conversion differ from that of the forward normal incidence. The back reflection of the down-conversion (red) is not shown here, because it follows the same energy/momentum conservation that leads to asymmetric photonic transitions. The optical path of the back reflection doesn't overlap with that of the forward case, and therefore the system is nonreciprocal. (**b**) Experimental demonstration of the nonreciprocal transition process for the forward and backward reflection from the dynamically modulated metasurface. In the forward reflection, a normal incident light (λ = 873 nm) hit the metasurface ($k_s = -0.48k_{probe}$). A dynamic phase modulation ($k_M = 0.31k_{probe}$ and $\Delta\omega$ = 2.8 THz) is imprinted upon the reflected light, leading to an up-conversion (λ = 865 nm, $k_x = -0.17k_{probe}$), and a down-conversion (λ = 881 nm and $k_x = -0.79k_{probe}$) whose reflection angle exceeds the effective collection angle of the lens (NA~ 0.76) and therefore cannot be detected. In the backward case, the time reversed counterpart of the up-conversion signal (λ = 865 nm and $k_x = 0.17k_{probe}$) is converted up to a mode with λ = 857 nm and $k_x = 0$, and down to a mode with λ = 873 nm and $k_x = -0.62k_{probe}$. None of the converted signals of the back reflection overlap with the forward incidence (λ = 873 nm and $k_x = 0$), therefore the back-reflection has a different trajectory from the forward-reflection and the system is nonreciprocal. In the dispersion diagram depicting the forward reflection, the converted signal is amplified by 10^5 for better illustration. The conversion signals are amplified by 10^6 in the backwards reflection, because the incident light hit the edge of the focusing lens, and therefore suffers from a poor spatial overlap with the travelling interference which lead to a lower conversion efficiency.

For the reason stated above, we focus on path (a). We show that the back reflection of the up-converted signal (or the anti-Stokes signal) generated in the forward propagation differs from the forward incidence both in frequency and reflection angle. In the forward propagation case (Fig. S8b), a normal incident light (λ = 873 nm) impinged on the metasurface ($k_s = -0.48\ k_{probe}$) with a dynamic phase modulation ($k_M = 0.31\ k_{probe}$ and $\Delta\omega$ = 2.8 THz). It experienced an anti-Stokes shift

(λ = 865 nm, k_x = -0.17 k_{probe}), and a Stokes shift (λ = 881nm and k_x = -0.79 k_{probe}). The Stokes signal had a reflection angle exceeding the effective collection angle of the lens (NA ~ 0.76) and therefore cannot be detected in our experiment. In the backward propagation case, we sent back the anti-Stokes signal (λ = 865 nm and k_x = 0.17 k_{probe}) onto the metasurface. It was partially up-converted to a mode of λ = 857 nm and k_x = 0, and partially down-converted to a mode λ = 873 nm and k_x = -0.62k_{probe}. Although the frequency of the down-converted signal coincides with that of original forward incidence, its transverse wavevector is different. Therefore, none of the back-converted signals go back to the state of the original forward incidence (λ = 873 nm and k_x = 0), and hence the system is nonreciprocal.

S5. Tunable dynamic modulation

As briefly mentioned in the main text, the optical dynamic modulation is very flexible, where $\Delta\omega$, k_M and k_s can be easily modified. Different $\Delta\omega$ can be obtained by varying the size of block attached to the center of the V-shaped split mirror. For instance, as shown in Fig. S9a, by using different size blocks, we get wavelength differences of 6 nm and 5 nm. Due to the experimental limitation, we have a limited range of $\Delta\omega$. However, we can realize a large and tunable $\Delta\omega$ with two different optical parametric amplifiers (OPA) [6], which can be used to build a tunable frequency-shifting optical isolator.

Meanwhile, k_M can be changed by adjusting the angle between the two pumps. Fig. S9b displays the Fourier transform analysis of interference patterns (insets) with varying k_M. It should be noted that the interference pattern is produced by the common frequency components in each pump, otherwise it would not be possible to observe the interference fringes because the pattern moves at high speed.

To demonstrate the importance of modifying k_M, we measured the dispersion diagram with $k_M = 0.23k_{probe}$ and $k_s = -0.48k_{probe}$ shown in Fig. S9c. It is clear that the reflected light experiences both upward and downward transitions because they both satisfy the phase matching condition and their tangential momenta are allowed in free space. By calculation, k_M must be at least $0.5k_{probe}$ for a normal incident input to achieve unidirectional photonic transitions in both forward and backward propagations. This is readily attainable by our experiment setup.

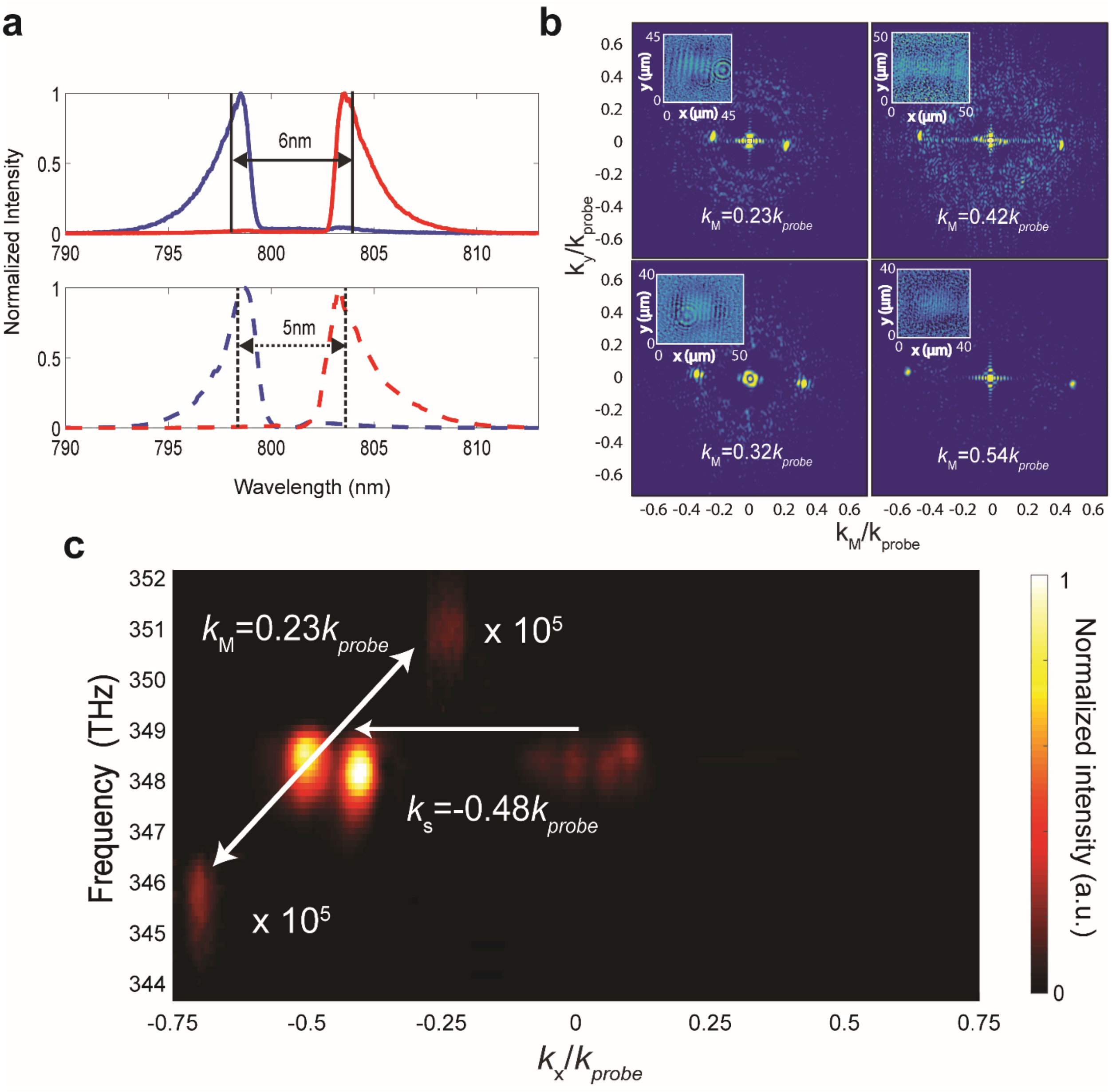

Fig. S9. Tunability of the optical dynamic modulation. (**a**) Pump beam spectra with central wavelength differences of 6 nm and 5 nm, respectively. (**b**) 2D Fourier transform of the interference patterns with various k_M. The insets show the interference pattern generated by the common frequency components in two pumps. (**c**) Metasurface ($k_s = -0.48k_{probe}$) modulated at $k_M = 0.23k_{probe}$ and $\Delta f = 2.8$ THz supports both upward and downward transitions. The converted signals are magnified by 10^5, and the experiment was carried out at peak pump intensity of 1 GWcm^{-2}.

In addition, we demonstrated arbitrary photonic transitions by using different k_M and k_s. As shown in Fig. S10 a-d, at fixed $k_s = 0.72\ k_{probe}$, the tangential wavevector of the down converted signal decreases with increased k_M, as determined by $k_x = k_s - k_M$. By the same token, when k_M is fixed at $0.31k_{probe}$, k_x can be changed by varying k_s (Fig. S10, e-g). This provides us with great flexibility in designing angle-shifting optical isolators.

We note that in Fig. S10 the conversion efficiency decreases from10^{-4} to 10^{-5} as k_M increases. Since the two pumps are separated further apart to create increased k_M, the focusing spot of the pumps is broadened as a result of the larger intersection angle between two pumps and distorted due to worse lens aberrations. Therefore, the peak pump intensity gradually decreases at the same input pump power, resulting in a reduced efficiency. Meanwhile, at larger k_M, the period of dynamic modulation decreases and covers fewer nanoantennas in a single period, which further decreases the efficiency. In addition, with the same k_M, the conversion efficiency increases as k_s increases. A larger k_s corresponds to a smaller supercell period; therefore more nanoantennas can be accommodated in a unit dynamic modulation period, which leads to a stronger modulation strength and higher efficiency.

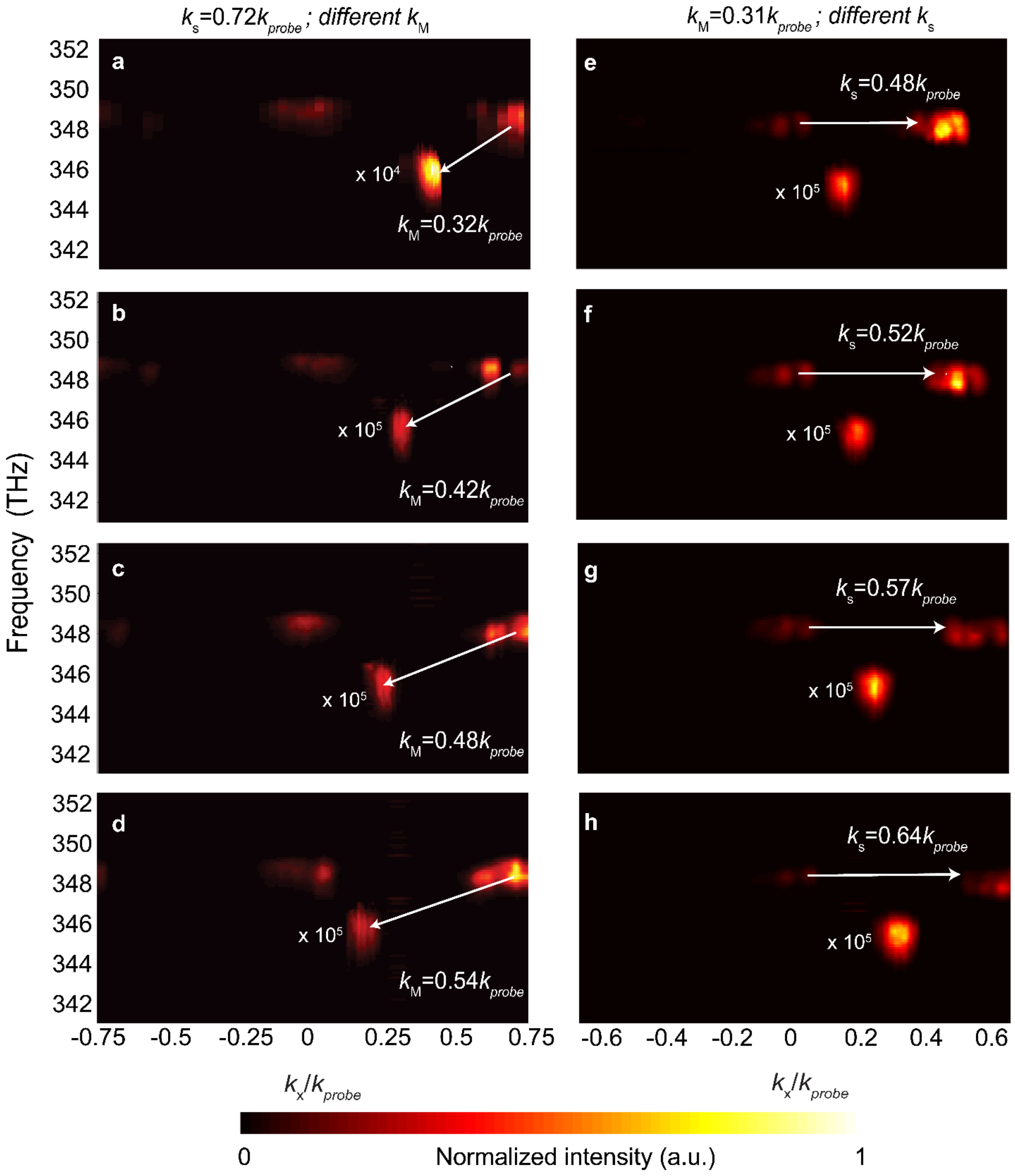

Fig. S10. k_s and k_M dependence of the nonreciprocal metasurface. (**a-d**) Dispersion diagrams of metasurface with k_s ranging from $0.48k_{probe}$ to $0.64k_{probe}$ at a fixed $k_M = 0.32k_{probe}$. This set of experiments were performed at the same total pump power of 320 mW. (**e-h**) At fixed $k_s = 0.72k_{probe}$, the dispersion diagrams of the metasurface modulated with k_M from $0.32k_{probe}$ to

$0.54k_{probe}$. This set of experiments were performed at a similar pump peak intensity of $1GWcm^{-2}$. The converted signals are magnified by 10^4 or 10^5 for better illustration.

S6. Analysis of the operational bandwidth of space-time metasurfaces

The bandwidth of time dependent nonreciprocal systems is usually determined by the phase matching conditions and modulation frequencies (more accurately, dynamic modulation induced frequency shift). For example, in ref. [7,8], bandwidths of a few hundred GHz were achieved by dispersion engineering at the expense of large device footprint, so that the phase matching condition was fulfilled over a larger bandwidth comparing with their counterparts[9]. It should be noted that this bandwidth is valid under monochromatic excitation. For broadband pulses the bandwidth is limited by the modulation frequency since we need to avoid overlap between the main pulse and its temporal-modulation-induced sidebands, which will cause signal distortion [10–12]. Our space-time metasurface works with optical modes in the continuum (not discrete guided modes) and naturally fulfills phase matching conditions in a broad frequency range. Besides, the meta-atoms in our system has a low quality factor (< 50) which allows the device to operate at a relatively broad bandwidth. In addition, our ultrafast modulation method features a modulation frequency $\Delta f \sim 2.8$ THz, which ensures at most 5.6 THz ($2\times \Delta f$) bandwidth with broadband excitation (Fig. S13). To sum up, with either a narrowband or broadband excitation, our metasurfaces exhibit several THz bandwidth, which is at least one order of magnitude greater than the largest ones(a few hundred GHz) [7,8] reported on time dependent nonreciprocal systems.

We measured the dispersion diagrams for both forward and backward reflections with a monochromatic probe ranging from 854 to 914 nm (Fig.S11). By fitting the converted signal efficiencies at different wavelengths, we obtained a − 3 dB bandwidth (full-width at half-maximum, FWHM) of ~5.77 THz (875 – 890 nm), which is determined by the broad optical resonance linewidth (Fig. S12). It should be noted that here we used a different sample from the one, which has degraded over time, used in the main text. The resonance of this sample shifted from 860 nm (Fig. S5) to 880 nm (Fig.S12). The backward reflection suffers lower conversion efficiency, because of the poor spatial overlap between probe and pump beams and the decreased diffraction efficiency of metasurface at oblique incidence. But these dispersion diagrams unequivocally show nonreciprocal reflections across a large wavelength range.

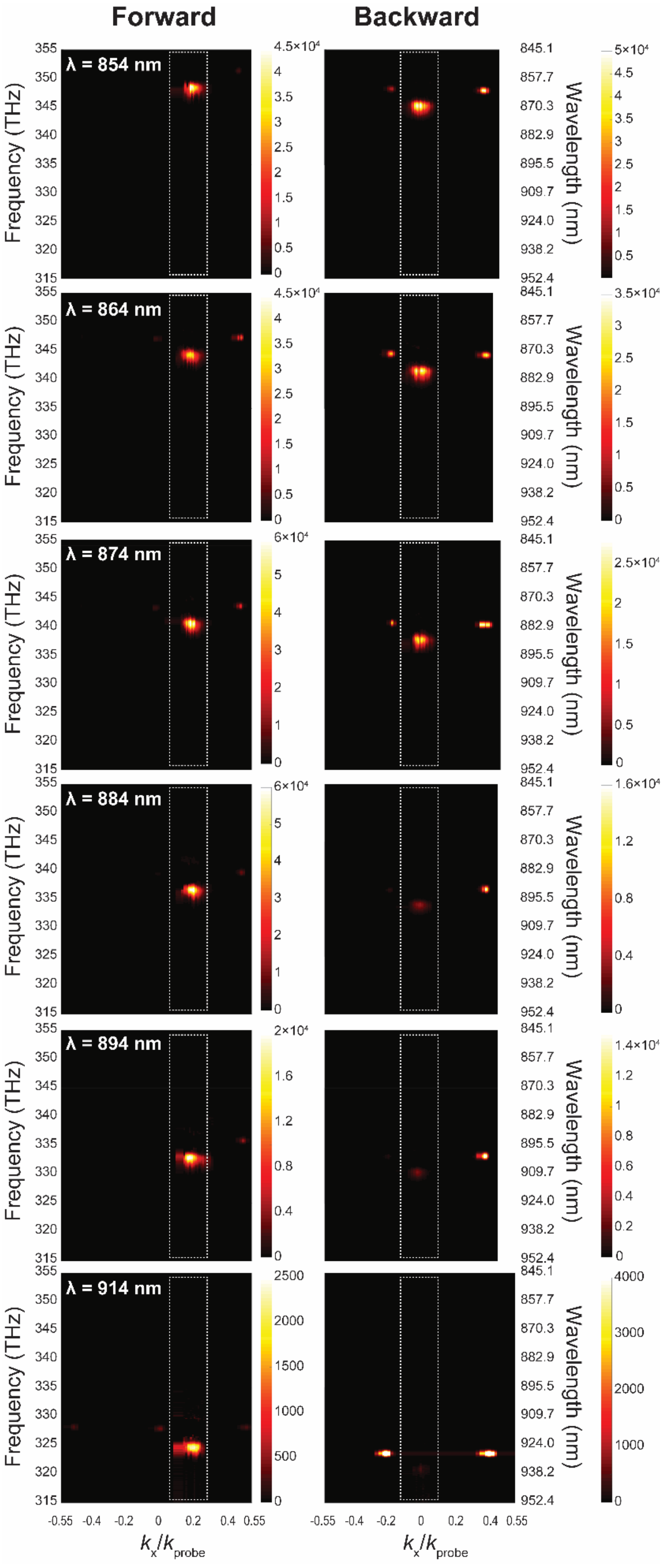

Forward
Backward
λ = 854 nm
λ = 864 nm
λ = 874 nm
λ = 884 nm
λ = 894 nm
λ = 914 nm
Frequency (THz)
Wavelength (nm)
k_x/k_{probe}

Fig. S11. Dispersion diagrams of nonreciprocal light reflections at probe wavelength from 854 nm to 914 nm. In the forward-propagating cases (figures in the left column), a normal incident light (with frequency f) was reflected by the metasurface, leading to a frequency down-shifted signal ($f - \Delta f$) with $k_x = k_{ms} - k_M$. In the backward-propagating cases (figures in the right column), the time-reversed counterpart of the signal was sent back and interacted with the metasurface again, leading to a reflected signal with frequency $f - 2\Delta f$ exiting at the normal direction. The dynamic modulation has $\Delta f = 2.8$ THz and $k_M = 0.35k_{probe}$ where $k_{probe} = 2\pi$ / (860 nm). The metasurface has a period of 1650 nm, corresponding to $k_{ms} = 2\pi$ / (1650 nm). The signals in the dashed white boxes are magnified by a factor of 4×10^5.

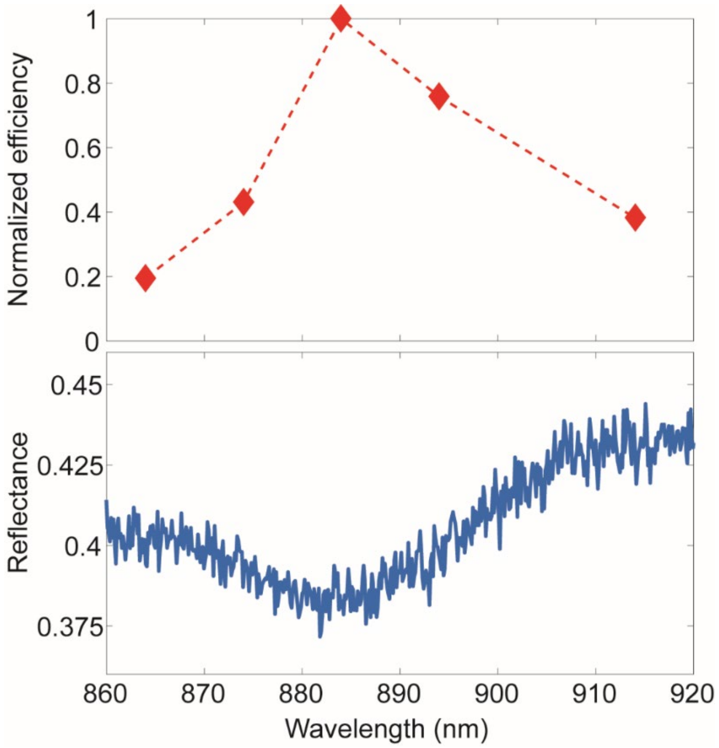

Fig. S12. Conversion efficiencies and linear reflectance spectra of the space-time metasurface experimented in Fig. S11. The normalized conversion efficiencies (top panel) show a similar response as the reflectance (bottom panel), which exhibit a broad resonance around 885 nm.

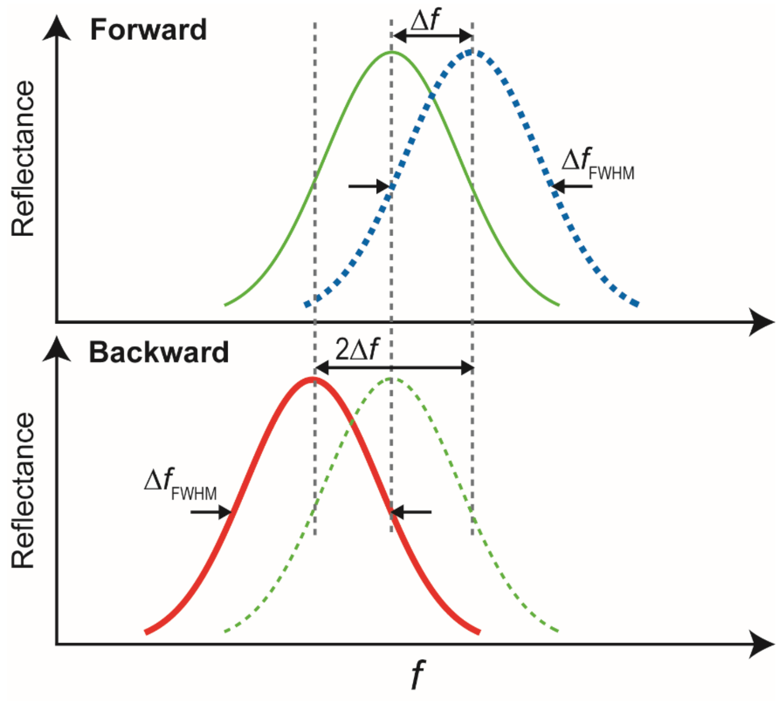

Fig. S13. Bandwidth of the nonreciprocal reflection of a broadband pulse incident on the space-time metasurface. The dashed lines represent the incident light, and the solid lines represent the reflected signals in both forward- and backward-propagating cases. As long as $2\Delta f$ (frequency shift between the forward incidence and the back-reflection) is larger than Δf_{FWHM} of the pulse, the back-reflection (solid red line) can be distinguished from the forward incident light (dashed blue line). Therefore, robust nonreciprocity is preserved with a bandwidth of at most $2\Delta f$.

S7. Analysis of the conversion efficiency and optimization methods

Roughly speaking, the first order conversion efficiency is proportional to $J_1(\Delta\varphi)^2$ according to our theory (equation (2)). We compared the efficiency at different peak pump intensities with theory as shown in Fig. S14. The efficiency was on the order of 10^{-4} with the pump intensity below the damage threshold of the samples. It started to deviate from the theoretical predication when the peak power intensity increases beyond 1 $\mathrm{GWcm^{-2}}$ using our high repetition rate (80 MHz) laser due to the possible thermal damages. Nevertheless, the conversion efficiency (dashed line) increases super-linearly with increasing peak power intensity, which leads to a tremendous boost in efficiency if pumped with a low-repetition-rate and high energy laser. In addition, the pulse width of the probe is stretched to 2 ps due to a series of nonlinear effects in the PCF. The focal spot of the probe is about 5 times larger than that of the pumps. Therefore, the converted signal

only comes from a small fraction of the incident probe. By improving the temporal and spatial quality of probe, the conversion efficiency can be further increased. Despite being limited by the experimental conditions, the conversion efficiency we achieved is still two orders of magnitude greater than the efficiency of the third-order nonlinear generation reported to date in amorphous silicon nanostructures at comparable pump intensities. [13] We also carried out a control experiment on a bare amorphous Si film but were not able to detect any converted signals, which confirmed that the amorphous Si resonant nanoantennas greatly enhanced the nonlinear induced dynamic phase change.

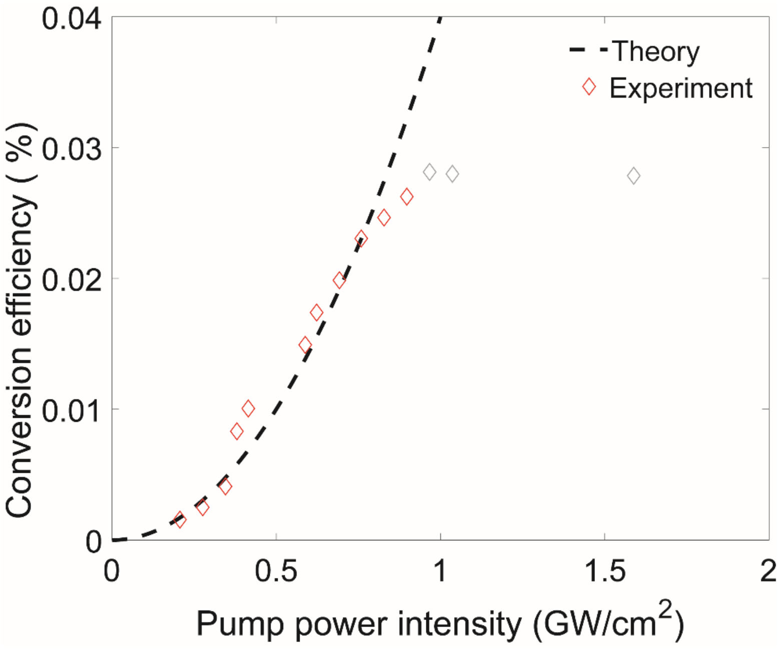

Fig. S14. Conversion efficiency of the nonreciprocal metasurface. The calculated and measured down-conversion efficiency on a sample with $k_s = 0.57k_{probe}$ and $k_M = 0.32k_{probe}$. They agree well with each other below the damage threshold (1 GWcm^{-2}) of the sample. At higher peak power intensity, the conversion efficiency saturates as the thermal effect permanently damage the sample.

In addition, as shown in our FDTD simulation results (Fig. S4), the conversion efficiency reaches up to 100% when $J_0(\Delta\varphi = 2.405)^2$ equals to zero, which is attainable with optimized nonlinear materials and metasurface designs. Here we propose to increase modulation depth and conversion efficiency with a low pump power requirement using the following methods.

First, we can use materials with large nonlinearity to construct the meta-atoms. It has been reported[14] that ITO in the ENZ region has extremely large nonlinear Kerr index that is around two orders of magnitude greater than that of amorphous silicon. A recent study[15] combined ITO thin film with gold nanoantennas and demonstrated even higher effective Kerr index (~ 3.73 cm^2GW^{-1}). To achieve a similar nonlinear phase shift (~ 0.03 radians) as demonstrated in our experiment, the required pump power intensity is below 100 $MWcm^{-2}$ with the ITO-antenna system. In addition, this ITO-nanoantenna system achieves a maximum nonlinear phase shift of 0.68 radians at pump intensity of 3.27 $GWcm^{-2}$ (energy density ~ 6.1 $pJ\mu m^{-2}$). According to equation (2) in our paper, the conversion efficiency can be:

$$\frac{J_1(\Delta\varphi)^2}{\sum_{n\in\{\text{all existent orders}\}} J_n(\Delta\varphi)^2} \times 100\% \approx 12\%$$

This conversion efficiency is about three orders of magnitude greater than that achieved in our experiment. Furthermore, by using high-quality-factor resonant nanoantennas and ENZ materials with low damping factors, the nonlinear phase shift can be further increased even at a moderate pump intensities[16,17]. It is worth noting that this conversion efficiency is realized with a subwavelength interaction length ~ 50 nm (total thickness of gold nanoantenna and ITO film). In comparison, a recent demonstration[8] on travelling modulation induced nonreciprocal system shows mode conversion efficiency of 1% at the cost of on-chip optical driving power of 90 mW and an interaction length of 2.39 cm.

Second, with further optimized meta-atom designs using amorphous silicon, we can get much larger phase modulation depth at lower pump intensity. Therefore, we are able to achieve same nonreciprocal effect with much lower pump power. We designed a new amorphous silicon meta-atom of which the working wavelength is around 1.55 μm. Our full-wave simulations show that we can achieve a very large phase shift change ($\Delta\varphi$) with a relatively low pump intensity (Fig. S15). Notably, at intensity of 0.4 $GWcm^{-2}$ (resulting $\Delta\varphi$ = 2.4 radians) with this new design, we achieve 100% mode conversion efficiency (no residual 0th order reflection remains). In addition, doubly resonant nanoantennas can be designed to have enhanced local field intensity at both pump and probe wavelengths, leading to a relaxed pump power requirement.

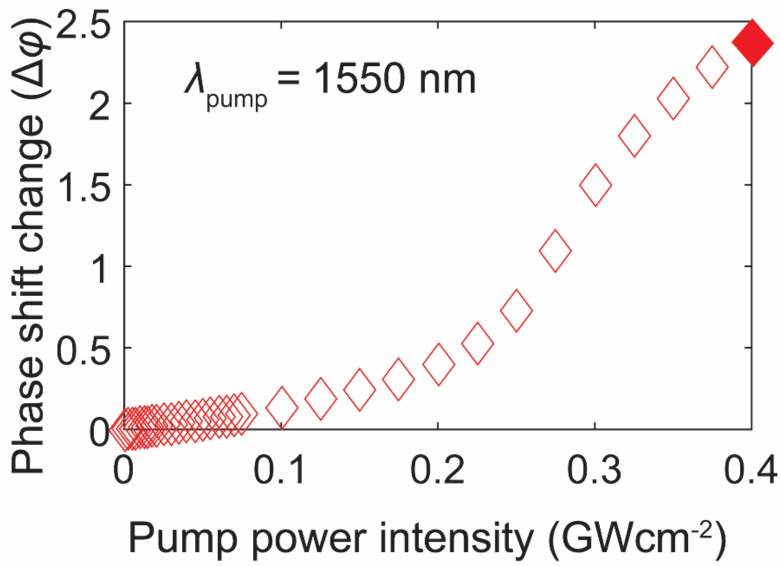

Fig. S15. Phase shift of change of a new meta-atom design. Full-wave simulations show phase shift change ($\Delta\varphi$) of a nonlinear amorphous silicon nanoantenna working at around 1.55 μm wavelength versus the pump intensity. The solid red diamond shows where 100% conversion efficiency can be achieved.

Last but not least, interaction time/length can be increased by stacking two space-time modulated metasurfaces to form a cavity. Photons are trapped inside the cavity to enable much longer interaction time with the travelling-wave modulation, leading to a boosted nonreciprocal conversion efficiency. In addition, a metasurface integrated resonator system can be used to increase the effective interaction length, For example, we can integrate metasurfaces on top of a micro-ring resonator, and apply a travelling wave modulation across the metasurface. Photons circulating inside the resonator will interact with the travelling-wave modulation for a time determined by the quality factor (Q-factor ~ 10^3 ~ 10^6) of the ring resonator, which is much larger than that of our current resonant antennas (Q-factor ~ 40).

References

1. Narayanan, K. & Preble, S. F. Optical nonlinearities in hydrogenated-amorphous silicon waveguides. *Opt. Express* **18**, 8998 (2010).

2. Shoji, Y. *et al.* Ultrafast nonlinear effects in hydrogenated amorphous silicon wire waveguide. *Opt. Express* **18**, 5668 (2010).

3. Shi, Y. & Fan, S. Dynamic non-reciprocal meta-surfaces with arbitrary phase reconfigurability based on photonic transition in meta-atoms. *Appl. Phys. Lett.* **108**, (2016).

4. Sounas, D. L., Estakhri, N. M. & Alù, A. Metasurfaces with engineered reflection and transmission: Optimal designs through coupled-mode analysis. in *2016 10th International Congress on Advanced Electromagnetic Materials in Microwaves and Optics, METAMATERIALS 2016* 346–348 (IEEE, 2016). doi:10.1109/MetaMaterials.2016.7746394

5. Shi, Y., Han, S. & Fan, S. Optical Circulation and Isolation Based on Indirect Photonic Transitions of Guided Resonance Modes. *ACS Photonics* **4**, 1639–1645 (2017).

6. Odoulov, S. *et al.* Interference and holography with femtosecond laser pulses of different colours. *Nat. Commun.* **6**, 5866 (2015).

7. Lira, H., Yu, Z., Fan, S. & Lipson, M. Electrically driven nonreciprocity induced by interband photonic transition on a silicon chip. *Phys. Rev. Lett.* **109**, 033901 (2012).

8. Kittlaus, E. A., Otterstrom, N. T., Kharel, P., Gertler, S. & Rakich, P. T. Non-reciprocal interband Brillouin modulation. *Nat. Photonics* **12**, 613–619 (2018).

9. Sohn, D. B., Kim, S. & Bahl, G. Time-reversal symmetry breaking with acoustic pumping of nanophotonic circuits. *Nat. Photonics* **12**, 91–97 (2018).

10. Kang, M. S., Butsch, A. & Russell, P. S. J. Reconfigurable light-driven opto-acoustic isolators in photonic crystal fibre. *Nat. Photonics* **5**, 549–553 (2011).

11. Sounas, D. L. & Alù, A. Non-reciprocal photonics based on time modulation. *Nat. Photonics* **11**, 774–783 (2017).

12. Maayani, S. *et al.* Flying couplers above spinning resonators generate irreversible

refraction. *Nature* **558**, 569–572 (2018).

13. Yang, Y. *et al.* Nonlinear Fano-Resonant Dielectric Metasurfaces. *Nano Lett.* **15**, 7388–93 (2015).

14. Alam, M. Z., De Leon, I. & Boyd, R. W. Large optical nonlinearity of indium tin oxide in its epsilon-near-zero region. *Science* **12**, 79–83 (2016).

15. Alam, M. Z., Schulz, S. A., Upham, J., De Leon, I. & Boyd, R. W. Large optical nonlinearity of nanoantennas coupled to an epsilon-near-zero material. *Nat. Photonics* **12**, 79–83 (2018).

16. Sachet, E. *et al.* Dysprosium-doped cadmium oxide as a gateway material for mid-infrared plasmonics. *Nat. Mater.* **14**, 414–420 (2015).

17. Caspani, L. *et al.* Enhanced Nonlinear Refractive Index in ε -Near-Zero Materials. *Phys. Rev. Lett.* **116**, 233901 (2016).
